# The Flagellin:Allergen Fusion Protein rFlaA:Betv1 Induces a MyD88− and MAPK-Dependent Activation of Glucose Metabolism in Macrophages

**DOI:** 10.3390/cells10102614

**Published:** 2021-10-01

**Authors:** Yen-Ju Lin, Garibald Papp, Csaba Miskey, Anna Fiedler, Alexandra Goretzki, Sonja Wolfheimer, Jennifer Zimmermann, Peter Crauwels, Zoltán Ivics, Ger van Zandbergen, Stefan Vieths, Stephan Scheurer, Stefan Schülke

**Affiliations:** 1 Vice Presidents Research Group 1: Molecular Allergology, Paul-Ehrlich-Institut, 63225 Langen, Germany; Yen-Ju.Lin@pei.de (Y.-J.L.); gpapp@students.uni-mainz.de (G.P.); A.Malczyk@gmx.de (A.F.); Alexandra.Goretzki@pei.de (A.G.); Sonja.Wolfheimer@pei.de (S.W.); Jennifer.Zimmermann@pei.de (J.Z.); Stefan.Vieths@pei.de (S.V.); Stephan.Scheurer@pei.de (S.S.); 2 Medical Biotechnology, Paul-Ehrlich-Institut, 63225 Langen, Germany; Csaba.Miskey@pei.de (C.M.); Zoltan.Ivics@pei.de (Z.I.); 3 Immunology, Paul-Ehrlich-Institut, 63225 Langen, Germany; petcrauwels@hotmail.com (P.C.); Ger.vanZandbergen@pei.de (G.v.Z.); 4 Institute of Immunology, University Medical Center of the Johannes Gutenberg University of Mainz, 55122 Mainz, Germany; 5 Research Center for Immunotherapy (FZI), University Medical Center, Johannes Gutenberg-University Mainz, 55122 Mainz, Germany

**Keywords:** flagellin, TLR5, allergen, Bet v 1, fusion protein, metabolism, Warburg, HIF-1a, immune metabolism

## Abstract

TLR5 ligand flagellin-containing fusion proteins are potential vaccine candidates for many diseases. A recombinant fusion protein of flagellin A and the major birch pollen allergen Bet v 1 (rFlaA:Betv1) modulates immune responses in vitro and in vivo. We studied the effects of rFlaA:Betv1 on bone marrow-derived macrophages (BMDMs). BMDMs differentiated from BALB/c, C57BL/6, TLR5^−/−^, or MyD88^−/−^ mice were pre-treated with inhibitors, stimulated with rFlaA:Betv1 or respective controls, and analyzed for activation, cytokine secretion, metabolic state, RNA transcriptome, and modulation of allergen-specific Th2 responses. Stimulation of BMDMs with rFlaA:Betv1 resulted in MyD88-dependent production of IL-1β, IL-6, TNF-α, IL-10, CD69 upregulation, and a pronounced shift towards glycolysis paralleled by activation of MAPK, NF*κ*B, and mTOR signaling. Inhibition of either mTOR (rapamycin) or SAP/JNK-MAPK signaling (SP600125) resulted in dose-dependent metabolic suppression. In BMDM and T cell co-cultures, rFlaA:Betv1 stimulation suppressed rBet v 1-induced IL-5 and IL-13 secretion while inducing IFN-γ production. mRNA-Seq analyses showed HIF-1a, JAK, STAT, phagosome, NLR, NF*κ*B, TNF, TLR, and chemokine signaling to participate in the interplay of cell activation, glycolysis, and immune response. rFlaA:Betv1 strongly activated BMDMs, resulting in MyD88−, MAPK−, and mTOR-dependent enhancement of glucose metabolism. Our results suggest macrophages are important target cells to consider during restauration of allergen tolerance during AIT.

## 1. Introduction

In the future, novel adjuvants and vaccines may be valuable tools to improve the efficacy, safety, and convenience of allergen immunotherapy. However, for their safe and efficient application, basic knowledge describing their immune modulating capacity, activated cell types, as well as a characterization of the underlying immunological mechanisms is needed.

The Toll-like receptor 5 (TLR5) ligand flagellin is a bacterial motility protein that forms the main body of the bacterial flagellum [1]. Because of its intrinsic immune-activating potential, flagellin was demonstrated to be an effective mucosal adjuvant mediating protective immune responses [2,3,4].

Since flagellin is the only proteinaceous TLR ligand, its application for the generation of recombinant flagellin:antigen fusion proteins is of special interest in vaccine development. Flagellin-containing fusion proteins have already been shown to be both safe and well-tolerated in clinical trials [5,6]. Consequently, fusion proteins combining flagellin with different antigens have been investigated for their potential to generate immune protection against different diseases including, among others: influenza [7,8,9], poxvirus [10], West Nile virus [11], and *C. tetani* [12], and *Pseudomonas* infections [13].

Moreover, flagellin:antigen fusion proteins are suggested as treatment options for IgE-mediated type I allergies [14,15,16,17]. Kitzmüller and colleagues recently described the enhanced immunogenicity, reduced allergenicity, and intrinsic adjuvant activity of flagellin C:Betv1 fusion proteins containing the major birch pollen allergen Bet v 1 in human monocyte-derived dendritic cells (DCs) and T cells from allergic patients [14].

In theory, adjuvant:antigen fusion proteins have the potential to both boost and modulate antigen-specific immune responses by targeting the fused antigen to immune cells in vivo that express receptors for the respective adjuvant. In addition, adjuvant-mediated immune cell activation influences the processing and increases the presentation of antigen-derived peptides in the context of the adjuvant-mediated immune cell activation [4]. Concordantly, our work has demonstrated that the fusion of allergens to the TLR5-ligand flagellin was able to suppress allergen-specific Th2 responses both in vitro and in vivo via the induction of both IL-10-mediated anti-inflammatory responses and Th1 responses [15,16,17,18]. However, the induction of overreaching Th1 responses may also have detrimental effects [19]. Therefore, the balanced induction of both pro- and anti-inflammatory responses by rFlaA:Betv1 may have advantages compared to exclusively triggering Th1-biased responses.

In our previous work, the fusion protein rFlaA:Betv1, consisting of flagellin A from *Listeria monocytogenes* and the major birch pollen allergen Bet v 1, was shown to suppress allergic sensitization in vivo [15]. These immune-modulatory effects were paralleled by a pronounced rFlaA:Betv1-mediated activation of myeloid dendritic cells (mDCs) in vitro, secretion of both pro- (IL-6, IL-12, and TNF-α) and anti-inflammatory (IL-10) cytokines, and a mammalian target of rapamycin (mTOR)-dependent metabolic switch towards glycolysis [15]. In this context, the contribution of both immune cell metabolism to the overall immune responses induced by such fusion proteins and other immune cell types needs further investigation.

Macrophages are both the main effector cells of the innate immune system and professional antigen-presenting cells (together with DCs and B cells). Therefore, macrophages control the induction and regulation of T cell immune responses via the uptake, processing, and presentation of antigens to antigen-specific T cells [20,21]. Epigenetics, cell survival, and the tissue microenvironment are the three main pathways controlling the phenotypical polarization of macrophages [22].

Based on both surface marker expression and their function, macrophages can be divided into classically activated (M1) or alternatively activated (M2) phenotypes [22]. Moreover, M2 macrophages can be further subdivided into the four sub-populations M2a, M2b, M2c, and M2d [23,24,25]. M1 macrophages are induced by either microbial products or Th1 cytokines, such as LPS, IFN-γ, or granulocyte-macrophage colony stimulating factor (GM-CSF) [22,26]. Functionally, they are capable of secreting pro-inflammatory cytokines, eliminating pathogens, and promoting a local Th1 environment [22,27].

M2a macrophage differentiation is induced by the Th2 cytokines IL-4 and IL-13; IL-1 receptor ligands and immune complexes were reported to induce M2b macrophages. IL- 10, TGFβ, and glucocorticoids were reported to induce M2c macrophages, and finally, TLR antagonists were reported to induce M2d macrophage differentiation [28]. Here, M2a macrophages secrete high levels of the Th2 cytokine IL-13 as well as other chemokines, which promote Th2 responses and induce eosinophil infiltration [28]. Both M2b and M2c macrophages are capable of secreting anti-inflammatory IL-10 in order to regulate immune responses. The M2d phenotype is known as a tumor-associated macrophage that regulates tumor metastasis [26]. While M1 and M2 macrophages are closely related to Th1 and Th2 responses, several studies have indicated the role of macrophages in allergic responses, especially in asthma [28,29,30,31,32]. In general, the M1 phenotype is the major effector macrophage in non-allergic asthma [31], and patients with higher frequencies of M1 cells have less severe clinical allergic reactions [29]. In contrast, M2 cells, especially of the M2a phenotype, are suggested to be the major effector macrophages in allergic asthma because they can promote Th2 responses, promote the secretion of histamine by interaction with antigen-specific Th2 cells, and induce eosinophil migration [28,29,30,31,32]. Therefore, understanding the effects of rFlaA:Betv1 on macrophages could further improve our knowledge of the immune-modulating properties of this promising vaccine candidate for type I allergies.

## 2. Materials and Methods

### 2.1. Generation of Recombinant Proteins

Recombinant flagellin A from *Listeria monocytogenes* (rFlaA, Acc. No: NC_003210) was generated according to [33]; recombinant birch pollen allergen Bet v 1 (Acc. No: X15877.1) was generated according to [34]. The fusion protein rFlaA and rBet v 1 (rFlaA:Betv1) was generated according to [15] by cDNA fusion using the cDNAs of both FlaA and Bet v 1 as templates. All proteins displayed an estimated purity greater than 95%, correct folding of secondary structure elements as determined by circular dichroism-spectroscopy, and an endotoxin content of 1.14 pg/µg protein (rFlaA), <0.48 pg/µg protein (rBet v 1), and 1.7 pg/µg protein (rFlaA:Betv1), respectively (data not shown).

### 2.2. Determination of Beta-Glucans

To exclude the immunological effects of TLR2 ligands in the used preparation of rFlaA:Betv1, the concentration of (1→3) beta-glucans contained within the used rFlaA:Betv1 preparation was determined using the Endosafe-PTS Portable Test System (Charles River Laboratories, Cologne, Germany) according to the manufacturer’s recommendations.

### 2.3. Mice

BALB/c, C57BL/6 mice, TLR5^−/−^, and MyD88^−/−^ mice (C57BL/6 background, all from Jackson Laboratories, Bar Harbor, Maine, ME, USA) were bred at the animal facility of the Paul Ehrlich Institute under specific pathogen-free conditions. All animal experiments were performed in compliance with the German animal protection law (granting authority: RP Darmstadt, Germany, Approval number: F107/131).

### 2.4. In Vitro Generation of Mouse Bone Marrow-Derived Macrophages, Stimulation, and Flow Cytometry

Mouse BMDMs were generated from bone marrow by culture in RPMI 1640 medium (Gibco, Karlsruhe, Germany) supplemented with 10% FCS, 1 mM sodium pyruvate, 10 mM HEPES, 100 U/mL penicillin, 100 µg/mL streptomycin, 1 mM l-glutamine, 0.1 mM 2-mercaptoethanol, and 20 ng/mL of mouse recombinant macrophage colony-stimulating factor (rmM-CSF, PeproTech, Hamburg, Germany). For differentiation into BMDMs, 2 × 10^6^ cells were seeded per 100 mm dish in 10 mL complete medium. On day 3, 10 mL of complete medium were added per culture dish. On days 6 and 8, 10 mL medium were exchanged and on day 10 M-CSF concentration was reduced to 12.5 ng/mL.

On day 11, BMDMs were harvested, seeded at 3.2 × 10^5^ cells/mL in 24-well plates (Thermo Scientific, Dreieich, Germany), and stimulated with the indicated equimolar concentrations of rFlaA, rBet v 1, rFlaA + rBet v 1, rFlaA:Betv1, or LPS for either 24 h or 96 h, based on the experimental design. As an additional control, BMDMs were stimulated with LPS amounts corresponding to the residual amounts contained within the rFlaA:Betv1 preparation used for BMDM stimulation. Supernatants were analyzed for cytokine secretion by ELISA using the following antibody combinations: IL-1β (capture antibody: IL-1β monoclonal mouse antibody 1:500 (#14-7012-85, eBioscience, Frankfurt, Germany) plus secondary detection antibody: IL-1β polyclonal mouse biotin-conjugated antibody 1:500 (#13-7112-81, eBioscience, Frankfurt, Germany)), IL-6 (capture antibody: IL-6 monoclonal mouse antibody 1:1000 (#14-7061-85, eBioscience) plus detection antibody: IL-6 monoclonal mouse biotin-conjugated antibody 1:1000 (#13-7062-85, eBioscience)), TNF-α (capture antibody: TNF-α monoclonal mouse antibody 1:500 (#14-7325-85, eBioscience) plus detection antibody: TNF-α monoclonal mouse biotin-conjugated antibody 1:250 (#13-7326-85, eBioscience)). Levels of IL-10 were measured using the mouse ELISA Development Kit from PeproTech (#900-T53, PeproTech) according to the manufacturer’s recommendations.

For flow cytometric analyses, BMDMs were seeded at 3.2 × 10^5^ cells/mL in 24-well plates (Thermo Scientific) and stimulated with the indicated equimolar concentrations of rFlaA, rBet v 1, rFlaA + rBet v 1, or rFlaA:Betv1 for 24 h. 10 µg/mL lipopolysaccharide (LPS, L5886, Sigma Aldrich, Taufkirchen, Germany) served as a positive control. Cells were stained with anti-mouse, Pacific Blue-conjugated CD11b (clone: M1/70.15, dilution: 1:50; Invitrogen, Thermo Fisher Scientific) and PE-Cy5-conjugated F4/80 (clone: BM8, dilution: 1 to 100, eBioscience). The activation of CD11b^+^F4/80^+^ BMDMs was assessed using anti-mouse PE-Cy7-conjugated CD69 (clone: H1.2F3, dilution: 1 to 100, eBioscience) and the PE-Cy7 intensity was quantified using a LSR II flow cytometer (BD Bioscience). For analysis of cell viability, BMDMs were treated as indicated and stained for dead cells using the fixable viability dye eFlour 780 (eBioscience). Data were analyzed using FlowJo V.7 (Treestar Inc., Ashland, OR, USA).

### 2.5. Preparation of BMDM:CD4^+^ T Cell Co-Cultures

BALB/c BMDMs were either cultured alone (5 × 10^5^ cells/mL) or with splenic CD4^+^ T cells (6.3 × 10^5^ cells/mL) isolated from BALB/c mice immunized with Bet v 1 plus alum (2 times 10 µg rBet v 1 plus 0.5 mg alum i.p., 2 weeks apart) using the CD4^+^ T Cell Isolation Kit (Miltenyi Biotech, Bergisch Gladbach, Germany). Cultures were stimulated with Bet v 1, rFlaA:Betv1, or rFlaA + rBet v 1 for 72 h. Subsequently, cytokine secretion into culture supernatants was determined using either BD OptEIA ELISA Sets (IFN-γ, IL-5, BD Bioscience), Ready-Set-Go-ELISA kits (IL-13, eBioscience) or the following antibody combination for IL-2: capture antibody: IL-2 monoclonal mouse antibody 1:500 (#503702, BioLegend, Koblenz, Germany) plus detection antibody: IL-2 monoclonal mouse biotin-conjugated antibody 1:500 (#503804, BioLegend).

### 2.6. Inhibitors

To analyze signaling pathways involved in rFlaA:Betv1-mediated BMDM activation, BMDMs were pre-incubated with the indicated amounts of rapamycin (mTOR inhibitor) or the respective MAPK inhibitors, including either SP600125 (SAP/JNK MAPK inhibitor), SB-202190 (p38α/β MAPK inhibitor, all Invivogen, Toulouse, France), or U0126 (MEK1/2 MAPK inhibitor, Cell Signaling Technologies, Leiden, The Netherlands) for 90 min and subsequently stimulated with rFlaA:Betv1 for either 24 h (ELISA) or 96 h (ELISA and analysis of cell metabolic state).

### 2.7. Western Blotting

For Western blot experiments, BMDMs were starved in RPMI1640 supplemented with 1% FCS (Sigma-Aldrich) and cultured for 3 h at 37 °C and 5% CO_2_ in either T25/75 flasks, cell culture plates, or FACS tubes. Subsequently, 1 × 10^6^ BMDMs were stimulated with the indicated proteins in RPMI1640. 30 min to 48 h post-stimulation, cells were washed with ice-cold PBS and subsequently lysed with 200 µL lysis buffer (62.5 mM Tris-HCl (pH 6.8), 2% w/v SDS, 10% glycerol, 50 mM DTT, 0.01% w/v bromophenol blue) for 10 min on ice. Target proteins in lysates were separated by SDS-polyacrylamide gel electrophoresis and transferred to nitrocellulose membranes. After blocking with 5% non-fat milk, the membranes were incubated with the following primary antibodies from Cell Signaling Technologies overnight at 4 °C: phospho-MAPK Family Antibody Sampler Kit (#9910), NF-κB Pathway Sampler Kit (#9936), mTOR Substrates Antibody Sampler Kit (#9862), Glycolysis Antibody Sampler Kit (#8337), anti-Phospho-Stat3 (Ser727) antibody (#9134), anti-HIF-1a antibody (#14179), anti-ACO2 antibody (#6571), and loading control anti-histone H3 antibody (#12648, HRP Conjugate) or anti-GAPDH antibody (#8884, HRP Conjugate). Detection was performed with the provided secondary antibodies using ACE Glow substrate (VWR, Darmstadt, Germany) and images were captured with either a Fusion-Fx7 Spectra reader (Vilber Lourmat, Eberhardzell, Germany) or an iBright CL1500 system (Thermo Fischer Scientific). Band intensities in Western blots were quantified with ImageJ software (imagej.nih.gov, version: 1.52a) as relative light units (RLU) normalized to the histone H3 loading control.

### 2.8. Analysis of Cell Metabolic State

The Warburg effect, glucose consumption, and metabolic rate in stimulated BMDM cultures were determined according to [15].

### 2.9. Metabolic Flux Analysis

For metabolic flux analysis, 1 × 10^5^ BMDMs per well were seeded in Seahorse XF96 cell culture microplates (V3-PS, TC-treated, Agilent, Santa Clara, CA, USA). The next day, the medium was exchanged, and cells were stimulated as indicated using the different proteins. Seahorse XF Real-Time ATP rate assays, Seahorse XF Glycolysis, and Seahorse XF Cell Mito Stress Tests were performed according to the manufacturer’s recommendations (Agilent). Cycle numbers were as follows: 4 cycles of baseline measurement, 14 cycles of stimulation with the different proteins, and 8 cycles with either oligomycin, rotenone/ antimycin A (Rot/AA), and 2-deoxy-glucose (2-DG), respectively (1 cycle = 3 min mixing plus 3 min measuring). Post-measurement, samples were normalized to total protein content via BCA (Thermo Fischer Scientific) and analyzed using Wave Desktop software (Agilent) and Graphpad Prism v8. Glycolytic Stress and Mito Stress results were analyzed using the respective report generator sheets according to the manufacturer’s recommendations (Agilent).

### 2.10. RNA-Seq and Bioinformatics

For RNA-Seq analyses, 3.2 × 10^5^ BMDMs/mL were stimulated in 24-well plates with either LPS as a positive control or the indicated equimolar concentrations of rFlaA + rBet v 1 or rFlaA:Betv1 for 48 h. Subsequently, BMDMs were harvested (4 biological replicates per condition) and frozen in Trizol (Invitrogen, Thermo Fisher Scientific) before total RNA isolation with the Direct-zol RNA Miniprep Kit (Zymo Research, Freiburg, Germany). We harnessed a modified version of the NNSR priming method [35] to prepare stranded, Illumina-compatible libraries as follows: we used the NEBNext Poly(A) mRNA Magnetic Isolation Module (NEB) to isolate mRNA from 1 µg total RNA per sample. cDNA was synthesized with Superscript IV in the presence of 4 µg Actinomycin D and RiboLock RNase inhibitor (Thermo Fisher Scientific) with the NNSR_RT primer (see Table 1 below) for 30 min at 45 °C, following the recommendations of the manufacturer. After subsequent magnetic bead purifications and RNase H (NEB) treatment, samples were subjected to second-strand cDNA synthesis with 3′-5′ exo(−) Klenow (NEB) and with the primer NNSR_2 for 30 min at 37 °C. The bead-purified DNA samples were amplified with the NNSRnest_ind_N and NNSR_Illumina primers using the NEBNext Ultra II Master Mix (NEB) to obtain the barcoded libraries with the following cycling conditions: 98 °C 10 s; 5 cycles of 98 °C 10 s, 55 °C 30 s, 68 °C 30 s; 15 cycles of 98 °C 10 s, 65 °C 30 s, 68 °C 30 s. The smears of PCR products were agarose gel-purified, and the libraries were sequenced on a NextSeq instrument with single-end 86 bp settings.

The raw sequencing reads were quality-, length-, and adapter-trimmed with *fastp* [36]. We used STAR [37] to align the trimmed reads to the human genome (hg38 assembly) and to count the mapped reads on gene level. The quantitative evaluation of the counts, differential gene expression, and the accompanying statistical analyses were performed in the R environment (https://www.r-project.org, version 3.5.0. (accessed on 23 April 2018) using the DESeq2 package (version 1.28.3)) [38]. KEGG pathway analysis [39] and gene set enrichment analysis were performed with the clusterProfiler package [40]. Data were deposited in the SRA database (accession number: PRJNA755840).

### 2.11. Statistical Analysis

Statistical analyses were performed with GraphPad Prism v6 to v8 for either Mac or Windows using 2-way ANOVA tests with confidence intervals adjusted for multiple comparisons according to either Bonferroni or Tukey. For statistically significant results, the following convention was used: * *p*-value < 0.05, ** *p*-value < 0.01, *** *p*-value < 0.001.

## 3. Results

### 3.1. Activation of BMDM Metabolism by rFlaA:Betv1 Is Accompanied by Both Pro- and Anti-inflammatory Cytokine Secretion

**Figure 1:**
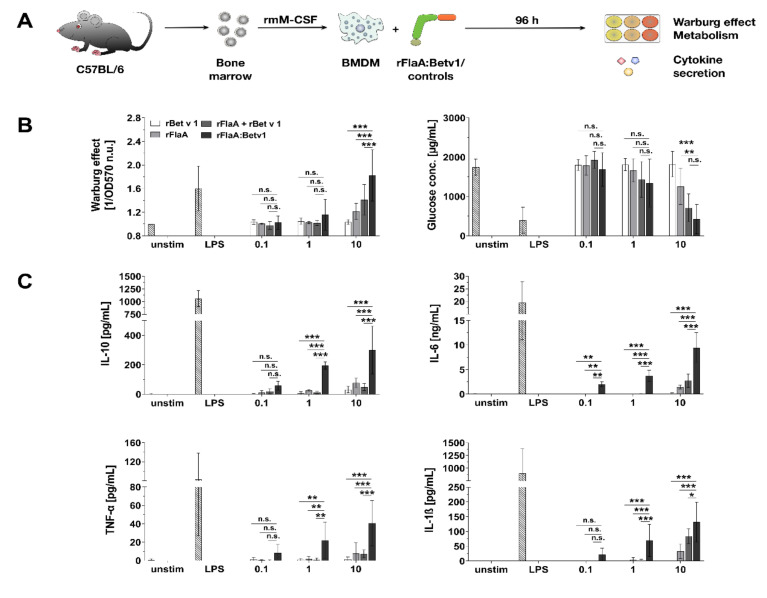
Activation of BMDM metabolism by rFlaA:Betv1 is accompanied by both pro- and anti-inflammatory cytokine secretion. C57BL/6 BMDMs were differentiated from mouse bone marrow and stimulated with the indicated equimolar protein amounts or LPS as a positive control for 96 h (**A**). Supernatants were analyzed for the induced Warburg effect and glucose consumption (**B**) as well as cytokine secretion by ELISA (**C**). Data are mean results of three independent experiments ± SD with two technical replicates per experiment. Statistical significance indicated as: n.s. *p*-value > 0.05, * *p*-value < 0.05, ** *p*-value < 0.01, *** *p*-value < 0.001.

BMDMs differentiated from C57BL/6 mice were stimulated with equimolar amounts of rBet v 1, rFlaA, the mixture of rFlaA and rBet v 1 (rFlaA + rBet v 1), or the fusion protein rFlaA:Betv1 for 96 h and analyzed for their cellular metabolic state and cytokine secretion profile (Figure 1A). Both rFlaA and rFlaA:Betv1 activated BMDM metabolism (Figure 1B). While differences between the rFlaA:Betv1- and either the rFlaA− or rFlaA + rBet v 1-induced Warburg effect were significant, there was no difference in glucose consumption between rFlaA:Betv1− and rFlaA + Bet v 1-stimulated BMDMs (Figure 1B). Activation of BMDM glucose metabolism was paralleled by a highly significantly increased production of both pro- (IL-1β, IL-6, TNF-α) and anti-inflammatory (IL-10) cytokines from rFlaA:Betv1-stimulated BMDMs compared to cells stimulated with either both proteins alone or provided as a non-fused mixture (for a stimulation concentration equimolar to 10 µg/mL rBet v 1, rFlaA:Betv1 induced 1.6-fold higher IL-1β secretion, 3.4-fold higher IL-6 secretion, 5.5-fold higher TNF-α secretion, and 6.1-fold higher IL-10 secretion than the respective mixture of both proteins, Figure 1C). Similar activation of BMDM metabolism (Repository Figure S1) and cytokine secretion profiles were observed for BALB/c BMDMs (Repository Figure S2).

To exclude potential immune-activating effects of the residual amounts of LPS contained within the used rFlaA:Betv1 preparation, we established thresholds for LPS-induced BMDM activation (Repository Figure S3A). Here, the threshold for an LPS-induced Warburg effect was between 1 and 10 ng. Increased glucose consumption and significant secretion of IL-10 were observed starting at 10 ng; secretion of IL-6 started at 1 ng, while IL-1β- and TNF-α-secretion were detected after stimulation with 1 µg of LPS (Repository Figure S3B,C). In addition, stimulation of BMDMs with the amounts of LPS contained within the used concentrations of rFlaA:Betv1 (0.59 pg LPS contained within 0.247 μg/mL rFlaA:Betv1, 5.9 pg LPS in 2.47 μg/mL rFlaA:Betv1, and 59 pg LPS in 24.7 μg/mL rFlaA:Betv1) neither resulted in activation of BMDM metabolism nor cytokine secretion (Repository Figure S3B).

To further exclude effects of LPS or potentially contaminating TLR2 ligands in the used rFlaA:Betv1 preparation, we used HEK293 cell lines stably transfected with either mouse TLR2, TLR4, or TLR5 as well as non-TLR-transfected HEK293 control cells (Repository Figure S4A). Here, rFlaA:Betv1 exclusively induced TLR5 activation, as indicated by a dose-dependent release of IL-8 from these cells (Repository Figure S4B). To further exclude the presence of TLR2 ligands in the rFlaA:Betv1 preparation, levels of (1→3) beta-glucans were determined. Detection of (1→3) beta-glucans in the used rFlaA:Betv1 preparation showed the levels of (1→3) beta-glucans to be below 0.8 pg/µg of protein, which is safely below the low ng amounts of different TLR2-ligands (LPS, mycoplasma diacylated lipoprotein, lipomannan, lipoarabinomannan, and Pam_3_CysK_4_) that were previously reported to activate TLR2 on BMDMs [41]. Therefore, we could show that neither the residual LPS amounts nor TLR2 ligands contribute to the rFlaA:Betv1-mediated activation of BMDMs.

In summary, stimulation of BMDMs with rFlaA:Betv1, and, to a lower degree, with rFlaA and rFlaA + rBet v 1, resulted in a pronounced activation of both BMDM metabolism and pro- and anti-inflammatory cytokine secretion.

### 3.2. rFlaA:Betv1 Triggers a Pronounced Shift Towards Glycolysis in BMDMs

**Figure 2:**
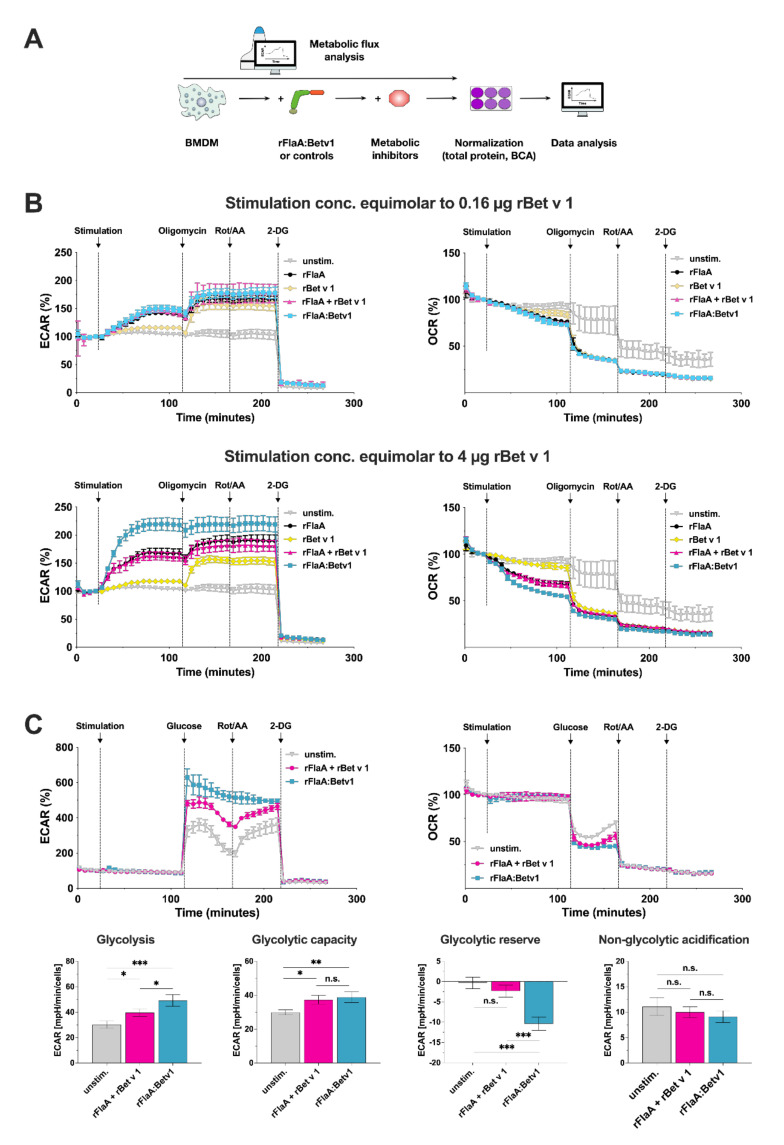
rFlaA:Betv1 triggers a pronounced shift towards glycolysis in BMDMs. Assay scheme for metabolic flux analysis using Seahorse technology (**A**). BMDMs were stimulated with either rFlaA + rBet v 1 or rFlaA:Betv1 in the indicated equimolar concentrations (0.16 μg rBet v 1 = 8 nM, and 4 μg rBet v 1 = 200 nM of protein) and analyzed for extracellular acidification rates (ECAR) and oxygen consumption rates (OCR) using Seahorse technology (**B**). 14 cycles (84 min) post-stimulation, ATP synthase, the electron transfer chain, and glycolysis were inhibited by sequential injection of oligomycin, rotenone/antimycin A (Rot/AA), and 2-deoxy-glucose (2-DG), for 8 cycles (48 min) each, respectively (**B**). Glycolytic stress test was performed in BMDMs stimulated with either rFlaA + rBet v 1 or rFlaA:Betv1 (both equimolar to 4 µg of rBet v 1) (**C**). Levels of glycolysis, glycolytic capacity, glycolytic reserve, and non-glycolytic respiration were analyzed by Seahorse technology using the “XF Glycolysis Stress Test Report Generator” according to the manufacturer’s recommendations (**C**). Data are representative results of three independent experiments (with three to four technical replicates per experiment) that showed similar results. Statistical significance indicated as: n.s. *p*-value > 0.05, * *p*-value < 0.05, ** *p*-value < 0.01, *** *p*-value < 0.001.

In-depth analysis of the BMDM metabolic state using Seahorse technology (Figure 2A) revealed rFlaA, rFlaA + rBet v 1, and rFlaA:Betv1 to dose-dependently increase glycolysis (detected by enhanced extracellular acidification rates (ECAR)), while at the same time reducing mitochondrial respiration (reduced oxygen consumption rates (OCR), Figure 2B). In contrast, rBet v 1 had only minimal effects on BMDM metabolism (Figure 2B). Further inhibition of the mitochondrial ATP synthase by oligomycin and of electron transport chain complexes by rotenone/antimycin A (Rot/AA) resulted in compensatory increases in ECAR, which were less pronounced in rFlaA:Betv1-stimulated BMDMs compared to the controls (Figure 2B). Treatment with the competitive inhibitor of glucose-6-phosphate, generation of 2-deoxy-glucose (2-DG) completely suppressed extracellular acidification (Figure 2B). In line with these results, analysis of the glycolytic state showed that rFlaA:Betv1-stimulated BMDMs have similar glycolytic capacities (capacity to produce energy by glycolysis) but higher glycolytic rates (amount of energy generated by glycolysis per minute and cell) compared to BMDMs stimulated with rFlaA + rBet v 1. In comparison, BMDMs stimulated with equimolar amounts of rFlaA + rBet v 1 have higher glycolytic reserves (capacity to further increase energy production by glycolysis) while no significant differences in non-glycolytic acidification (acidification of the extracellular medium by processes other than glycolysis) were observed between treatment groups (Figure 2C). In contrast to this, detailed analysis of mitochondrial function via Mito Stress tests revealed no differences in basal respiration, spare respiratory capacity, mitochondrial ATP production, proton leak, and non-mitochondrial respiration between rFlaA + rBet v 1- and rFlaA:Betv1-stimulated cells (Repository Figure S5).

Taken together, the metabolic phenotyping of rFlaA:Betv1-stimulated BMDMs suggested the fusion protein induced a pronounced shift towards increased levels of glycolysis while mitochondrial respiration was suppressed.

### 3.3. rFlaA:Betv1-Induced BMDM Metabolism and Inflammatory Cytokine Secretion Depend on MyD88 While Only Being Partially TLR5-Dependent

**Figure 3:**
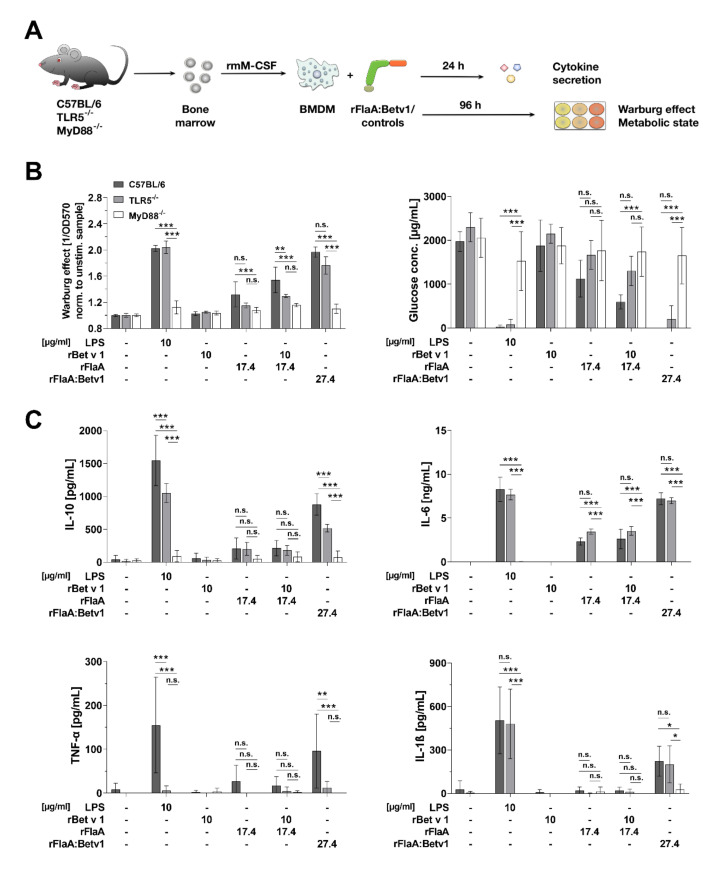
rFlaA:Betv1-induced BMDM metabolism and inflammatory cytokine secretion depend on MyD88 while being only partly TLR5-dependent. C57BL/6, TLR5^−/−^, or MyD88^−/−^ BMDMs were differentiated from mouse bone marrow and stimulated with the indicated equimolar protein amounts or LPS as a positive control for either 24 h (cytokine secretion, **C**) or 96 h (metabolic state, **B**) (**A**). Supernatants were analyzed for the induced Warburg effect and glucose consumption (**B**) as well as cytokine secretion by ELISA (**C**). Data are mean results of three independent experiments ± SD with two technical replicates per experiment. Statistical significance indicated as: n.s. *p*-value > 0.05, * *p*-value < 0.05, ** *p*-value < 0.01, *** *p*-value < 0.001.

In order to study the signaling mechanisms contributing to rFlaA:Betv1-mediated BMDM activation, BMDMs were differentiated from either C57BL/6, TLR5-, or MyD88-deficient mice, stimulated with the fusion protein and the respective controls, and analyzed to assess their metabolic state and cytokine secretion profile (Figure 3A).

Interestingly, activation of BMDM metabolism (Warburg effect and glucose consumption) by both rFlaA and rFlaA:Betv1 was shown to be partially TLR5-dependent (Figure 3B). MyD88-deficient BMDM did not show increased metabolic activity after stimulation with either of the tested proteins (Figure 3B). In line with these results, cytokine secretion induced by either rFlaA, rFlaA + rBet v 1, or rFlaA:Betv1 was shown to be abrogated in MyD88-deficient BMDMs (Figure 3C). Among the tested cytokines, only rFlaA:Betv1-induced IL-10 and TNF-α showed a substantial TLR5 dependency (41% and 87% reduction in TLR5-deficient mice compared to C57BL/6 BMDMs, respectively) while secretion levels of all other cytokines were unaffected by TLR5 deficiency (Figure 3C).

In summary, using BMDMs differentiated from either TLR5− or MyD88-deficient bone marrow, the activation of glucose metabolism and cytokine secretion was shown to be abrogated in the absence of MyD88 while being partially TLR5 dependent for the secretion of IL-10 and TNF-α.

### 3.4. rFlaA:Betv1 Induces MAPK, NF*κ*B, and mTOR Signaling in BMDMs

**Figure 4:**
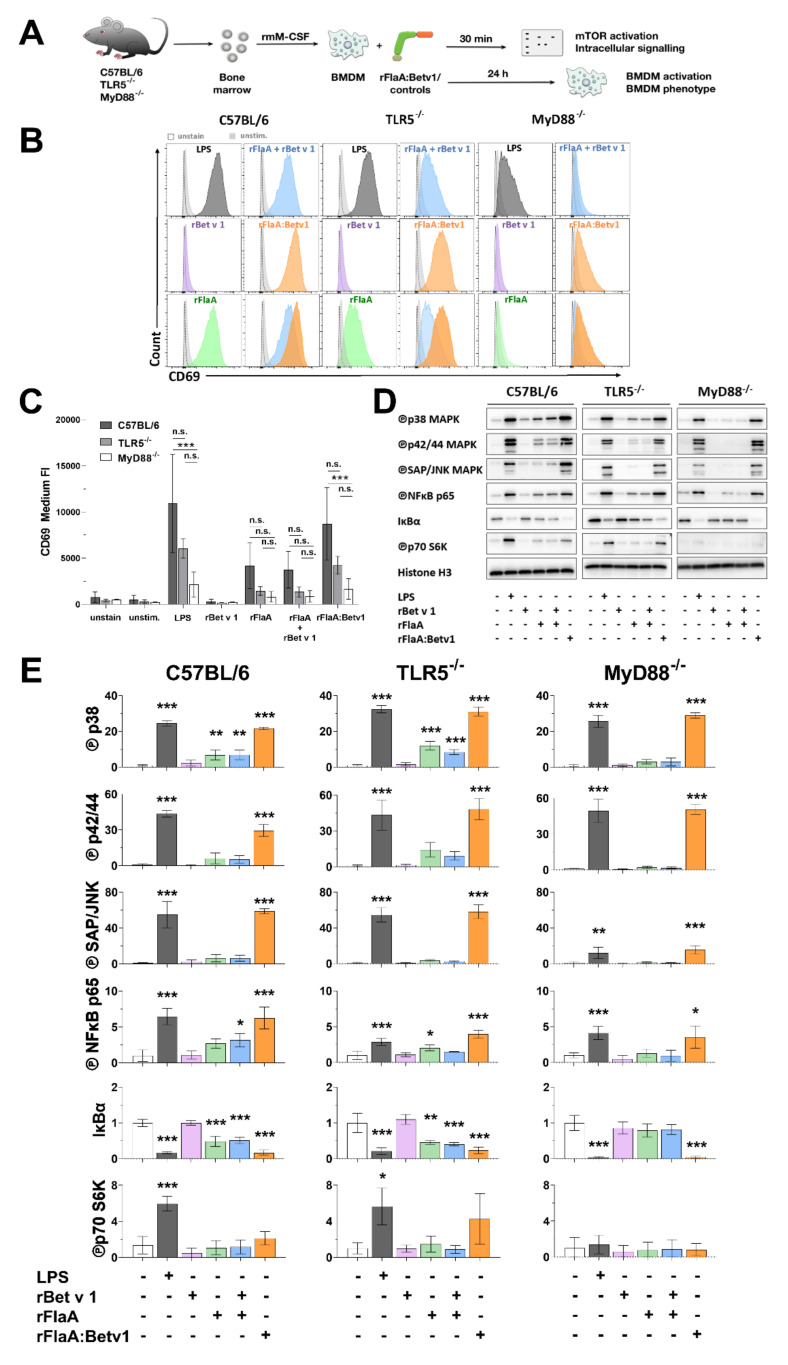
rFlaA:Betv1-stimulation induces stronger BMDM activation characterized by signaling of MAPK, NF*κ*B, and mTOR **.** C57BL/6, TLR5^−/−^, or MyD88^−/−^ BMDMs were differentiated from mouse bone marrow and stimulated with the indicated equimolar protein amounts or LPS as a positive control for either 30 min (Western blot, **D**) or 24 h (flow cytometry, **B**,**C**) (**A**). Expression levels of CD69 were analyzed by flow cytometry (exemplary result in **B**, mean fluorescence intensity of three independent experiments depicted in **C**) and activation of MAPK, NF*κ*B, and mTOR1 signaling was analyzed by Western blot (**D**). Western blots from three independent experiments were quantified and normalized to expression levels of histone H3 (**E**). Indicated are statistically significant differences compared to unstimulated controls. Data are either representative results from three independent experiments (**B**,**D**) or mean results of three independent experiments ± SD (**C**,**E**) with either 10.000 BMDMs (**B**,**C**) or one lysate (**D**,**E**) measured per experiment. Statistical significance indicated as: n.s. *p*-value > 0.05, * *p*-value < 0.05, ** *p*-value < 0.01, *** *p*-value < 0.001.

Subsequently, the activation status and intracellular signaling cascades of C57BL/6, TLR5^−/−^, or MyD88^−/−^ BMDMs stimulated by rFlaA:Betv1 were analyzed (Figure 4A).

Stimulation of C57BL/6 BMDMs with rFlaA:Betv1 resulted in higher up-regulation of CD69 compared to either rFlaA− or rFlaA + rBet v 1-stimulation (Figure 4B and quantified in Figure 4C). Of note, both rFlaA− and rFlaA:Betv1-induced CD69 upregulation was partially, but not significantly, reduced in TLR5-deficient BMDMs, while being completely abrogated in MyD88-deficient BMDMs (Figure 4B,C). Stimulation with rBet v 1 alone in either tested mouse strain did not result in increased CD69 expression (Figure 4B,C).

Stimulation of C57BL/6 BMDMs with rFlaA:Betv1 resulted in strongly increased levels of phosphorylated MAP kinases p38, p42/44, and SAP/JNK, phosphorylation of NF*κ*B p65, as well as phosphorylation of the mTOR target protein p70 S6 kinase compared to either rBetv 1−, rFlaA−, or rFlaA + rBet v 1-stimulated BMDMs (Figure 4D,E, left panel). Here, both rFlaA− and rFlaA:Betv1-induced phosphorylation of MAP kinases, NF*κ*B, and p70 S6 kinase was reduced but still detectable in TLR5-deficient BMDMs (Figure 4D,E, middle panel). Interestingly, while rFlaA-induced phosphorylation of p70 S6 kinase and MAP kinases was abrogated in MyD88-deficient BMDMs, rFlaA:Betv1-induced MAP kinase, SAP/JNK, and NF*κ*B activation could still be detected in MyD88-deficient BMDMs (Figure 4D,E, right panel).

Taken together, we observed rFlaA:Betv1 to induce a stronger activation of MAPK, NF*κ*B, and mTOR signaling in BMDMs compared to either rFlaA alone or the mixture of both proteins. rFlaA:Betv1-induced activation of MAPK and NF*κ*B signaling was largely TLR5− and MyD88-independent, while p70 S6 kinase activation was only abrogated in MyD88-deficient BMDMs.

### 3.5. Activation of BMDM Metabolism and IL-10 Secretion are Partly mTOR-Dependent while Pro-Inflammatory Cytokine Secretion Is mTOR-Independent

**Figure 5:**
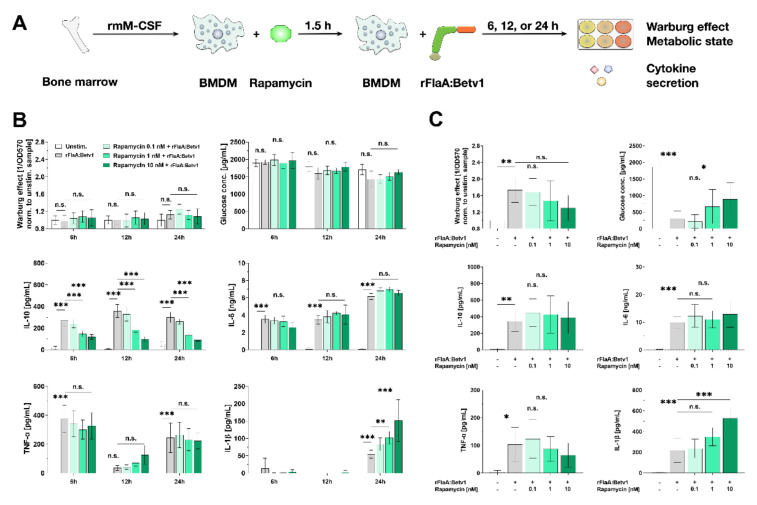
rFlaA:Betv1-induced activation of C57BL/6 BMDM metabolism and IL-10 secretion are partly mTOR-dependent, while pro-inflammatory cytokine secretion is mainly mTOR-independent. C57BL/6 BMDMs were differentiated from mouse bone marrow, pre-treated with the indicated rapamycin (mTOR inhibitor) concentrations for 90 min, and subsequently stimulated with rFlaA:Betv1 for an additional 6, 12, 24, or 96 h (**A**). Cells were analyzed for their metabolic state and cytokine secretion by ELISA after either 6 to 24 h (**B**) and 96 h (**C**). Data are mean results of three independent experiments ± SD, with two technical replicates per experiment. Statistical significance indicated as: n.s. *p*-value > 0.05, * *p*-value < 0.05, ** *p*-value < 0.01, *** *p*-value < 0.001.

**Figure 6:**
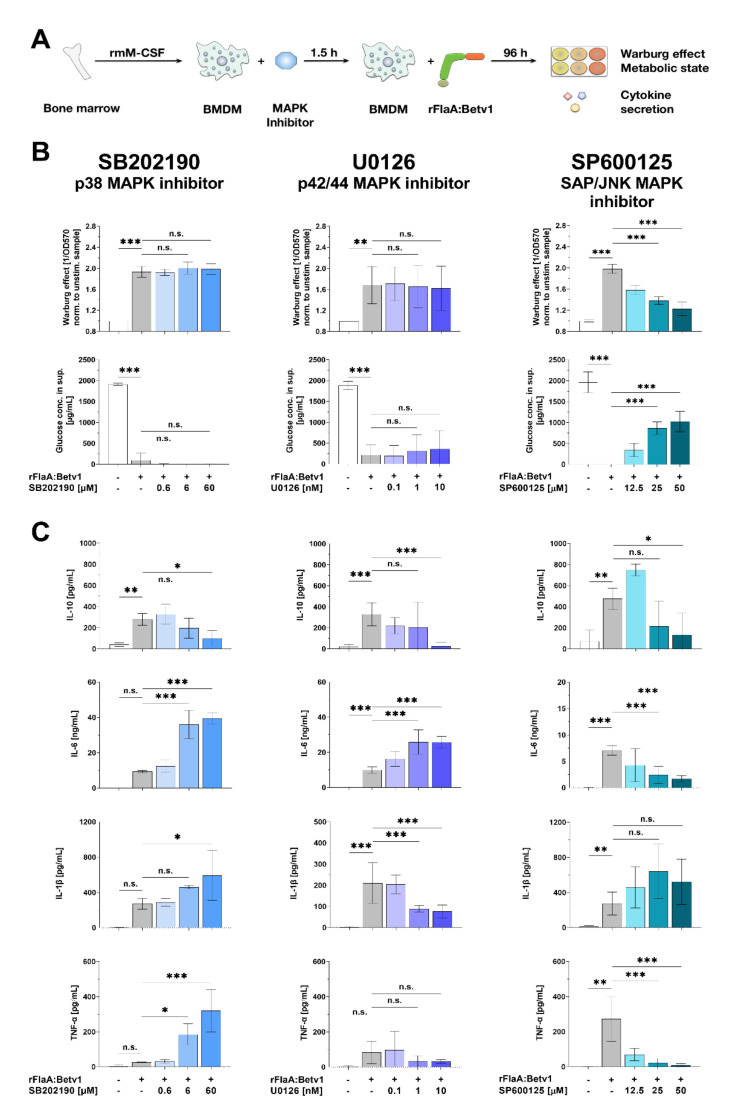
SAP/JNK MAP kinase signaling contributes to rFlaA:Betv1-induced activation of BMDM metabolism as well as both pro- and anti-inflammatory cytokine secretion. C57BL/6 BMDMs were differentiated from bone marrow, pre-treated with the indicated MAPK inhibitors (either SB202190 (p38 MAPK inhibitor), U0126 (p42/44 MAPK inhibitor), or SP600125 (SAP/JNK MAPK inhibitor)) for 90 min, and subsequently stimulated with rFlaA:Betv1 for additional 96 h (**A**). Cells were analyzed for their metabolic state (**B**) and cytokine secretion by ELISA (**C**). Data are mean results of three independent experiments ± SD, with two technical replicates per experiment. Statistical significance indicated as: n.s. *p*-value > 0.05, * *p*-value < 0.05, ** *p*-value < 0.01, *** *p*-value < 0.001.

To study the contribution of either mTOR and/or different MAP kinases to rFlaA:Betv1-mediated activation of BMDM metabolism and cytokine secretion, BMDMs were pre-incubated with either rapamycin to inhibit mTOR activation (Figure 5A) or different MAPK inhibitors (Figure 6A). Toxicity of all tested inhibitors on BMDMs was excluded by live/dead staining (Repository Figure S6).

To inhibit mTOR complex 1 (mTORC1) activation, BMDMs were pre-treated with rapamycin and checked for the Warburg effect, glucose consumption, and cytokine secretion after either 6, 12, 24, or 96 h (Figure 5B,C). Interestingly, dependent on the time, the engagement of mTOR had different effects on IL-10 secretion.

While up to 24 h post-stimulation a rFlaA:Betv1-induced Warburg effect and increased glucose consumption were not yet detectable, mTOR inhibition dose-dependently suppressed rFlaA:Betv1-induced IL-10 secretion at all investigated time points. Here, pro-inflammatory cytokine secretion was either unaffected (IL-6 & TNF-α) or even increased (IL-1β, Figure 5B). In contrast to this, at 96 h post-stimulation, inhibition of mTORC1 activation slightly, but not significantly, inhibited the rFlaA:Betv1-induced Warburg effect and reduced glucose consumption from the culture medium (Figure 5C). With the exception of a dose-dependent 2.4-fold increase in rFlaA:Betv1-induced IL-1β secretion, rFlaA:Betv1-induced IL-10, IL-6, and TNF-α secretion were all unaffected by mTOR-inhibition (Figure 5C).

To compensate for a potential degradation of rapamycin in the BMDM cultures, we added 2.5 nM of rapamycin daily for 4 days and compared the results to a one-time application of 10 nM rapamycin (as was done before in Figure 5, Repository Figure S7A). Here, in accordance with our previous results, neither a single application of 10 nM rapamycin at the beginning of the culture nor sequential adding of rapamycin over the whole culture time had any significant effects on either the rFlaA:Betv1-induced Warburg effect, glucose consumption, or cytokine secretion (Repository Figure S7B).

Therefore, these data suggest that, while rFlaA:Betv1-induced pro-inflammatory cytokine secretion is largely mTOR-independent, early rFlaA:Betv1-induced IL-10 secretion is mediated via mTOR. At later time points, mTOR-independent factors seem to drive IL-10 secretion.

### 3.6. MAP Kinase Signaling Contributes to rFlaA:Betv1-Induced Activation of BMDM Metabolism as well as Both Pro- and Anti-Inflammatory Cytokine Secretion

To investigate the contribution of different types of MAPK to rFlaA:Betv1-induced activation of BMDM metabolism and cytokine secretion, BMDMs were pre-treated with either the p38 MAPK inhibitor SB202190, the p42/44 MAPK inhibitor U0126, or the SAP/JNK MAPK inhibitor SP600125 (Figure 6). Interestingly, inhibition of different types of MAP kinases had differential effects on rFlaA:Betv1-induced activation of BMDM metabolism and cytokine secretion (Figure 6A).

Inhibition of p38 MAPK by pre-treatment with SB202190 and p42/44 MAPK by pre-treatment with U0126 had no effect on either the rFlaA:Betv1-induced Warburg effect or glucose consumption (Figure 6B, left and middle lane). In contrast to this, inhibition of SAP/JNK MAPK by SP600125 dose-dependently and highly significantly suppressed the rFlaA:Betv1-induced Warburg effect and glucose consumption (Figure 6B, right lane).

Inhibition of all types of MAPK dose-dependently inhibited rFlaA:Betv1-induced IL-10 secretion (Figure 6C). IL-6 secretion was significantly increased upon inhibition of either p38 MAPK (4.2-fold increase compared to rFlaA:Betv1-stimulated cells) or p42/44 MAPK (2.6-fold increase), but inhibited upon SAP/JNK MAPK-inhibition (4.1-fold reduction) (Figure 6C). rFlaA:Betv1-induced IL-1β secretion was significantly increased upon inhibition of either p38 MAPK inhibition (2.2-fold reduction compared to rFlaA:Betv1-stimulated cells) or SAP/JNK MAPK inhibition (1.9-fold increase), but significantly suppressed upon p42/44 MAPK inhibition (2.7-fold reduction) (Figure 6C). Finally, rFlaA:Betv1-induced TNF-α secretion was 12.6-fold increased by pre-treatment with the p38 MAPK inhibitor SB202190, while SAP/JNK MAPK inhibition (28.9-fold) suppressed TNF-α production (Figure 6C).

In summary, MAPK activation was shown to significantly contribute to rFlaA:Betv1-mediated BMDM activation. While the tested MAPK inhibitors differentially influenced rFlaA:Betv1-mediated cytokine secretion, only SAP/JNK MAPK activation was shown to be involved in the activation of glucose metabolism. Statistical significance indicated as: n.s. *p*-value > 0.05, * *p*-value < 0.05, ** *p*-value < 0.01, *** *p*-value < 0.001;

### 3.7. rFlaA:Betv1 Suppresses Th2 Responses from Bet v 1-specific CD4^+^ T Cells In Vitro

**Figure 7:**
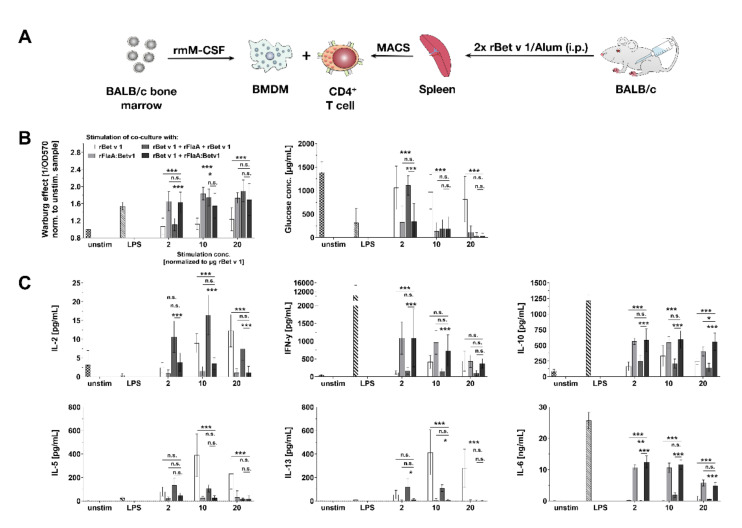
rFlaA:Betv1 suppresses rBet v 1-specific Th2 responses in vitro. BALB/c BMDMs were differentiated from mouse bone marrow, co-cultured with CD4^+^ T cells isolated by magnetic activated cell sorting (MACS) from rBet v 1 plus Alum-sensitized mice and stimulated with the indicated equimolar protein amounts or LPS as a positive control for 96 h (**A**). Supernatants were analyzed for their metabolic state (**B**) and cytokine secretion by ELISA (**C**). Data are mean results of three independent experiments ± SD with two technical replicates per experiment. Statistical significance indicated as: n.s. *p*-value > 0.05, * *p*-value < 0.05, ** *p*-value < 0.01, *** *p*-value < 0.001.

To test the immune-modulatory capacity of rFlaA:Betv1-stimulated BMDMs, cells were cultured with CD4^+^ T cells isolated ex vivo from rBet v 1 plus alum-sensitized BALB/c mice (Figure 7A). Co-cultures were re-stimulated with either (I) rBet v 1 to induce rBet v 1-specific recall responses, (II) equimolar amounts of rFlaA:Betv1 to check for rFlaA:Betv1-induced metabolic changes and cytokine secretion in BMDM:CD4^+^ T cell co-cultures, (III) rBet v 1 plus the equimolar mixture of rFlaA + rBet v 1 to analyze the effect of the protein mixture on rBet v 1-induced cytokine responses, or (IV) rBet v 1 plus rFlaA:Betv1 to investigate the effect of the fusion protein on rBet v 1-induced cytokine responses.

Interestingly, stimulation with rBet v 1 only induced a very slight Warburg effect in BMDM:CD4^+^ T cell co-cultures, which was stronger after stimulation with the mixture of rFlaA + rBet v 1 (equimolar to 10 µg rBet v 1), while rFlaA:Betv1 induced a strong Warburg effect. even in the lowest stimulation concentration (Figure 7B). Results of glucose consumption paralleled the results observed for the Warburg effect (with high Warburg effects resulting in high glucose consumption, Figure 7B).

rBet v 1-induced IL-2 secretion was further boosted upon co-stimulation with the mixture of both proteins, while being suppressed after co-stimulation with rFlaA:Betv1 (Figure 7C). Stimulation of co-cultures with either rFlaA:Betv1 or rBet v 1 + rFlaA:Betv1 resulted in significantly increased levels of IFN-γ or IL-6 secretion (Figure 7C). Here, both levels of rFlaA:Betv1-induced IFN-γ and IL-6 secretion were reduced for the highest stimulation concentration (equimolar to 20 µg rBet v 1/mL) compared to the other stimulation concentrations (Figure 7C). In line with previous results, stimulation of BMDM:CD4^+^ T cell co-cultures with rFlaA:Betv1 resulted in pronounced anti-inflammatory IL-10 secretion (Figure 7C). Here, levels of secreted IL-10 were approximately 500 pg/mL for all tested stimulation concentrations (Figure 7C). rBet v 1-induced secretion of the Th2 cytokines IL-5 and IL-13 was significantly suppressed by either co-stimulation with either rFlaA + rBet v 1 or rFlaA:Betv1 (Figure 7C). Of note, in contrast to rBet v 1, rFlaA:Betv1 did not induce secretion of Th2 cytokines IL-5- and IL-13 from BMDM:CD4^+^ T cell co-cultures (Figure 7C). Neither rFlaA + rBet v 1 nor rFlaA:Betv1 directly activated the metabolism or cytokine secretion from naïve CD4^+^ T cells (data not shown).

Taken together, rFlaA:Betv1-mediated activation of BMDM metabolism was also observed in BMDM:CD4 T cell co-cultures. Here, both addition of rFlaA:Betv1 and rFlaA + rBet v 1 suppressed the rBet v 1-induced secretion of Th2 cytokines, while rFlaA:Betv1 also triggered significant production of IFN-γ, IL-6, and IL-10.

### 3.8. rFlaA:Betv1 Induces a Transcriptional Shift Towards HIF-1a-Mediated Glycolytic Metabolism in BMDMs

**Figure 8:**
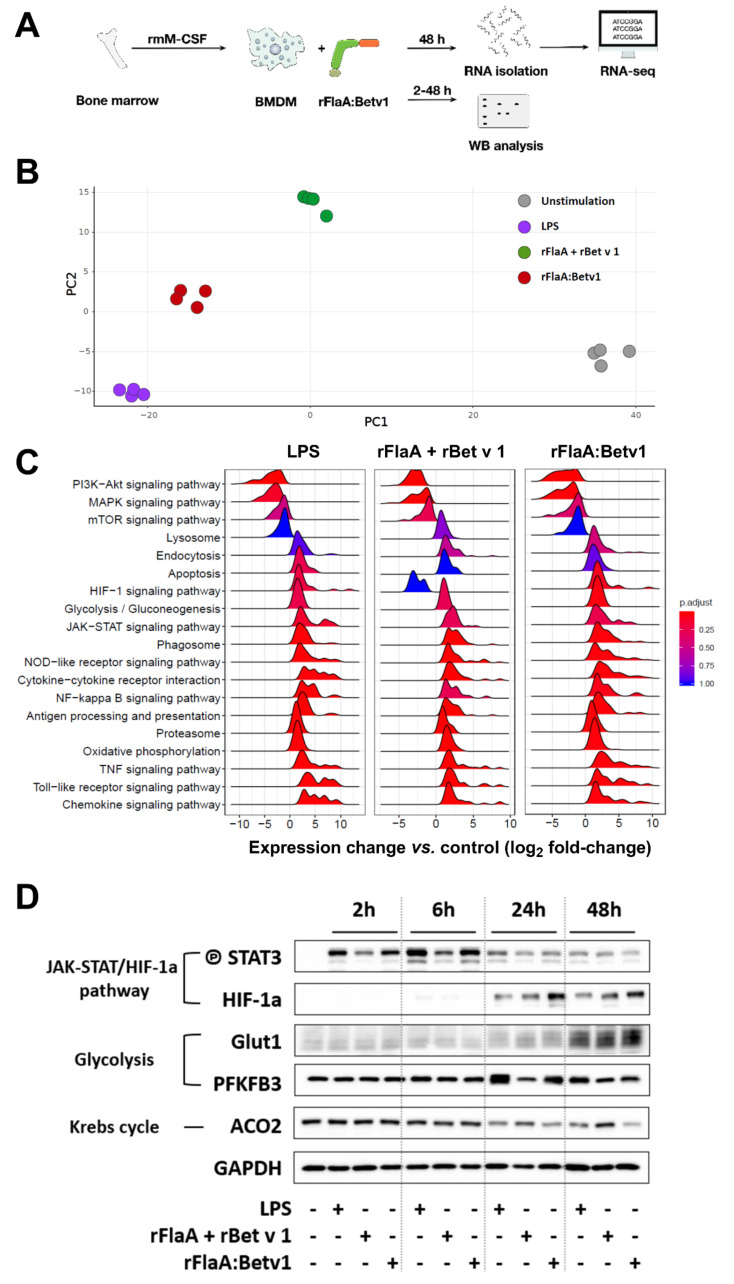
rFlaA:Betv1 induces a transcriptional shift towards HIF-1a-mediated glycolytic metabolism in BMDMs. C57BL/6 BMDMs were differentiated from mouse bone marrow and stimulated with either LPS as a positive control or equimolar amounts of either rFlaA + rBet v 1 or rFlaA:Betv1 for 2 to 48 h (**A**). Cells were harvested and used for either RNA-seq (**B**,**C**) or Western blot analyses (**D**). To characterize the transcriptional status of the different samples, principal component analysis (**B**) and gene set enrichment analysis of the most significantly regulated KEGG pathways (**C**) was performed. BMDMs stimulated for 2, 6, 24, or 48 h with either LPS, rFlaA + rBet v 1, or rFlaA:Betv1 were analyzed by Western blot for expression levels of the indicated molecules (**D**). Data are either mean (**C**) or representative (**D**) results of three to four independent experiments using either one RNA preparation (**B**,**C**) or one lysate (**D**) per experiment.

To (I) confirm both the activation of glycolytic metabolism and BMDM activation and (II) investigate the regulation of other signaling pathways in rFlaA:Betv1-stimulated BMDMs in an unbiased way, BMDMs were stimulated with either LPS, rFlaA + rBet v 1, or rFlaA:Betv1 for 48 h, and whole-cell RNA expression profiles were generated using MiSeq technology (Figure 8A). In addition, samples were collected for Western blot analysis 2, 6, 24, and 48 h post-stimulation (Figure 8A).

Principal component analyses showed the biological replicates for each stimulation condition to closely group with each other, while the different stimulation conditions formed clearly separated groups (Figure 8B). Here, rFlaA:Betv1-stimulated BMDMs showed similar cell activation capacity as LPS-stimulated cells (Figure 8B).

The most prominently regulated KEGG pathways for the different stimulation conditions are depicted in Figure 8C. In general, an upregulation of endocytosis, glycolysis, antigen processing and presentation, proteasome, oxidative phosphorylation, and signaling of HIF, JAK-STAT, phagosomes, NLR, NF*κ*B− TNF, TLR, and chemokines was observed 48 h post-stimulation. Instead, signaling pathways mediating the early events of BMDM activation, such as phosphatidylinositol 3 kinase (PI3K)-AKT, MAPK, and mTOR signaling, were down-regulated compared to unstimulated samples (Figure 8C). Here, a general similarity between LPS- and rFlaA:Betv1-stimulated samples was again observed (Figure 8C). Compared to rFlaA + rBet v 1-stimulated BMDMs, rFlaA:Betv1-stimulation resulted in an upregulation of pathways associated with HIF-1a, TNF, TLR, and chemokine signaling, while lysosome-associated signaling was downregulated (Figure 8C).

Finally, activation of JAK-STAT-HIF-1a signaling, glycolysis, and the downregulation of mitochondrial respiration observed in transcriptomic samples were confirmed by Western blot (Figure 8D). Here, we observed a phosphorylation of STAT3 2, 6, and 24 h post-stimulation, which was more pronounced in LPS− and rFlaA:Betv1-stimulated BMDMs, and followed by a strong upregulation of HIF-1a starting 24 h post-stimulation after the peak of STAT3 phosphorylation at 6 h (Figure 8D). We also observed a stronger expression of the glucose transporter Glut-1 in all stimulation conditions 48 h post-stimulation and a stronger expression of the glycolysis-promoting enzyme 6-phosphofructo-2-kinase/fructose-2,6-biphosphatase 3 (PFKFB3) in LPS− and rFlaA:Betv1-stimulated BMDMs 24 h post-stimulation. In accordance with the observed switch to glycolytic metabolism, expression levels of the mitochondrial enzyme aconitase 2 (ACO2) were lower in LPS− and rFlaA:Betv1-stimulated BMDMs both 24 and 48 h post-stimulation (Figure 8D).

Taken together, the results obtained by RNA-seq analyses confirm a pronounced shift towards anaerobic metabolism in rFlaA:Betv1−, LPS−, and, to a lesser extent, rFlaA + rBet v 1-stimulated BMDMs.

**Figure 9:**
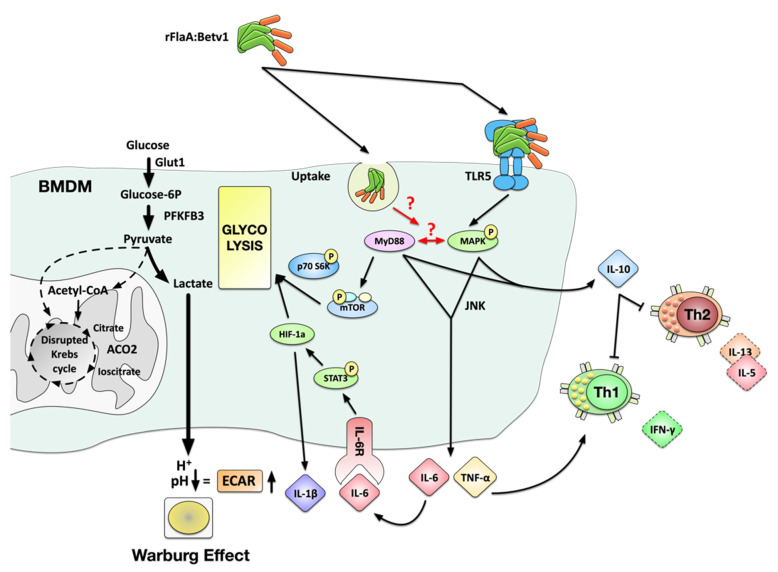
Suggested molecular signaling events contributing to rFlaA:Betv1-mediated activation of BMDMs. Stimulation of BMDM with aggregated rFlaA:Betv1 results in both TLR5-dependent and -independent uptake, leading to the activation of both MyD88− and MAPK-mediated signaling cascades. rFlaA:Betv1-induced pro-inflammatory IL-6 and TNF-α secretion were shown to be MyD88− and MAPK-dependent, likely promoting the induction of IFN-γ-producing Th1 cells in BMDM:TC co-cultures. Western blot analysis confirmed rFlaA:Betv1 to trigger STAT3 activation, increasing the expression of HIF-1a, which is likely to be responsible for the observed IL-1β secretion. Metabolically, we found a MyD88-dependent phosphorylation of the mTOR target protein p70 S6 kinase, which together with HIF-1a likely mediated the observed shift towards enhanced glycolysis with increased levels of the glucose transporter Glut-1 and PFKFB3, and the induction of the Warburg effect while being paralleled by a decreased expression of mitochondrial proteins such as ACO2 and reduced oxygen consumption rates, suggesting a disrupted Krebs cycle in these cells. Finally, TLR5, MyD88, and MAPK activation were shown to be involved in the rFlaA:Betv1-induced production of the anti-inflammatory cytokine IL-10 that likely caused the observed suppression of rBet v 1-induced Th1 and Th2 responses in BMDM:TC co-cultures.

Our current mechanistic understanding of the molecular events contributing to rFlaA:Betv1-mediated BMDM activation is depicted in Figure 9.

## 4. Discussion

In the present study, we analyzed the activation, metabolic state, and transcriptional state of BMDMs, as well as their immune-modulatory properties upon stimulation with a flagellin:allergen conjugate (rFlaA:Betv1), which was recently shown in preclinical studies to prevent allergic Th2 responses [15]. Here, the allergen Bet v 1 serves as a model antigen that is conjugated to the TLR5 ligand flagellin FlaA, which acts as an adjuvant activating the immune system. Thereby, FlaA may modulate the immunological properties of the conjugated antigen while also targeting the antigen to innate immune cells.

Currently, the effects of flagellin-containing fusion proteins on macrophages, which serve as important innate immune cells, are less described. So far, only one study by Verma et al. has described the insertion of a cytochrome C molecule into the flagella of *Salmonella dublin* to efficiently trigger processing and presentation of cytochrome-derived peptides by mouse peritoneal macrophages [42]. This prompted us to investigate the effect of FlaA and FlaA-containing fusion proteins on mouse macrophages.

### 4.1. rFlaA:Betv1 Induces a Partly TLR5-Dependent Activation of BMDMs Characterized by Both Pro- and Anti-Inflammatory Cytokine Secretion

We observed a stronger activation of mouse BMDMs by rFlaA:Betv1 compared to the respective controls. However, both stimulation with rFlaA and rFlaA + rBet v 1 resulted in a weaker, but still clearly detectable, activation of cytokine secretion and glycolytic metabolism in BMDMs. In line with the available literature [2,3,4], these results confirmed the adjuvant effect of non-conjugated flagellin.

BMDM activation by rFlaA:Betv1 was characterized by strongly increased pro- (IL-1β, IL-6, TNF-α) and anti-inflammatory (IL-10) cytokine secretion. In terms of the net outcome of this mixed pro- and anti-inflammatory response, the observed suppression of rBet v 1-induced Th2 cytokine secretion from BMDM:CD4^+^ T cell co-cultures suggested rFlaA:Betv1-mediated anti-inflammatory signaling (at least in higher stimulation concentrations) to be more pronounced.

While rFlaA:Betv1-mediated TNF-α secretion was largely TLR5-dependent, IL-10 secretion was partially reduced in TLR5^−/−^ BMDMs, and IL-1β and IL-6 secretion were found to be TLR5-independent. In line with our results, Bao and colleagues reported *S. typhimurium* FliC to induce a TLR5-dependent TNF-α production via the activation of PI3K/AKT /mTOR-dependent NF*κ*B and STAT3 signaling in mouse macrophages [43]. Inhibition of both PI3K by either LY294002 or wortmannin and the mTOR complex 1 (mTORC1) by rapamycin decreased flagellin-induced TNF-α and IL-6 expression as well as macrophage proliferation [43]. In an additional study, flagellin-induced TNF-α production from alveolar macrophages [44] was shown to be TLR-dependent, while FliC-induced IL-1ß secretion from mouse macrophages was shown to be IPAF-dependent, but TLR5-independent [45]. Interestingly, upregulation of CD69 was substantially reduced in TLR5^−/−^ BMDMs, while neither rFlaA:Betv1-induced cytokine secretion nor cell activation were observed in MyD88-deficient BMDMs.

Interestingly, we observed a significantly increased secretion of IL-1β upon inhibition of mTOR in rFlaA:Betv1-stimulated BMDMs both 24 and 96 h post-stimulation. Tang et al. reported similar results from tuberous sclerosis complex 1 (TSC-1)-deficient macrophages, which display constitutively active mTOR signaling [46]. Here, impaired (LPS-induced) IL-1β secretion from tuberous sclerosis complex 1 (TSC-1)-deficient macrophages could be increased by either inhibition of mTOR activation or mTOR deletion [46]. Mechanistically, mTOR-mediated downregulation of the CCAAT enhancer-binding protein (C/EBPβ) was shown to critically contribute to the reduction in IL-1β production [46]. These results suggest that, both in the model of Tang et al. and in rFlaA:Betv1-stimulated BMDMs, TSC-1 may promote IL-1β production via the C/EBPβ pathway, while activation of mTOR suppresses both TSC-1 itself and TSC-1-dependent downstream processes (including IL-1β production) [46].

In our previous publication describing the molecular characterization of rFlaA:Betv1, we reported rFlaA:Betv1 to be aggregated [15]. Here, rFlaA:Betv1 formed high molecular aggregates, which were only visible under native PAGE conditions [15]. Increased uptake of these aggregates in myeloid dendritic cells was shown to be TLR5-independent while being essential for the induction of rFlaA:Betv1-mediated IL-10 and IL-6 secretion [15]. Therefore, aggregation was previously shown to be an important factor determining the immunogenicity of the fusion proteins.

Mechanistically, we assume the stronger BMDM-activating potential of the fusion protein compared to both single proteins to result from: (i) the previously observed aggregation of rFlaA:Betv1 [15], which results in higher densities of the fusion protein on the surface of BMDMs, higher protein uptake, and therefore stronger activating signals transmitted to the respective BMDMs nucleus, (ii) changes in BMDM metabolic state due to the strongly activating signal provided by rFlaA:Betv1, and (iii) (for the induction of adaptive immune responses by rFlaA:Betv1-stimulated BMDMs) differences in antigen processing upon fusion to flagellin already reported for rOva contained within a rFlaA:Ova fusion protein [18].

Taken together, these results suggest that both TLR5-dependent and -independent processes contribute to rFlaA:Betv1-mediated BMDM activation, while MyD88 is a key molecule in rFlaA:Betv1-mediated macrophage activation.

### 4.2. rFlaA:Betv1-Stimulated BMDMs Efficiently Modulate Allergen-Specific T Cell Responses

Considering that the activation of BMDMs by rFlaA:Betv1 is antigen unspecific, we checked for the effect of rFlaA:Betv1-stimulated BMDMs on Th2-biased Bet v 1-specific T cells in an ex vivo co-culture model. Here, both rFlaA + rBet v 1 and rFlaA:Betv1 efficiently suppressed rBet v 1-induced Th2-cytokine secretion, while only rFlaA:Betv1 boosted IL-10 and IFN-γ secretion, once again confirming the pronounced immune modulatory capacity and Th1-inducing capacity of the fusion protein. One weakness of the employed co-culture setup is that the cellular source of the secreted cytokines (BMDMs or T cells) cannot be exactly determined. However, despite this limitation, our data provide evidence that simultaneous targeting of adjuvant and antigen to BMDMs efficiently promotes modulation of antigen-specific effector T cell responses. While in this study we investigated rBet v 1-specific T cell responses, our previous work on the model antigen ovalbumin [16,18] and the major mugwort allergen Art v 1 [17] suggests that the findings can also be transferred to other antigens.

### 4.3. rFlaA:Betv1 Activates Both Glycolytic Metabolism and HIF-1a Signaling in BMDMs

A further objective of this study was to explore the mechanisms underlying the observed immune modulation in more detail. Previous reports suggested mTOR to be engaged in rFlaA:Betv1-mediated cytokine responses [15]. rFlaA:Betv1 also triggered changes in the metabolic phenotype of the stimulated BMDMs. In activated immune cells, distinct metabolic changes not only provide the energy needed by these cells to proliferate and fulfill their effector functions, but are also used to generate important immunological active effector molecules (e.g., prostaglandins, ROS, NOS, or itaconate generated from a disrupted Krebs cycle in highly glycolytic cells) [47]. Therefore, cellular metabolism and signaling pathways classically associated with the activation and effector function of immune cells have recently been recognized to be intimately intertwined, regulating each other, and thereby shaping the overall immune responses induced.

We observed a highly significant induction of the Warburg effect in rFlaA:Betv1-stimulated BMDMs. The observed induction of the Warburg effect suggests a cellular shift towards glycolysis with lactate generation and extracellular acidification [48]. The suggested metabolic changes were confirmed using both Seahorse Metabolic Flux and transcriptomics analyses. RNA Seq confirmed the activation of both BMDM glycolytic metabolism (upregulation of key glycolysis enzymes Glut-1/2, phospho-fructo-kinase (PFK), PFKFB3, phosphoglycerate mutase (PGM), pyruvate kinase isozyme (PKM), and pyruvate dehydrogenase kinase 1 (PDK1)), intracellular signaling (TLR, MAPK, and NF*κ*B signaling), and both cytokine and chemokine secretion (Repository Figure S8). Taken together, the activation status and metabolic phenotype of rFlaA:Betv1-stimulated BMDMs closely relates to M1-polarized macrophages, which switch their metabolism towards glycolysis after being activated by pathogen-associated molecular patterns [49,50,51,52,53]. Increases in glycolytic metabolism enable M1-like macrophages to efficiently fulfill their effector function, including the production of pro-inflammatory cytokines [54] and phagocytosis [55].

In our experimental setting, HIF-1a, which was shown to be essential for glycolytic metabolism in macrophages, seems to be of particular importance. HIF-1a-deficient macrophages lack both lactate and ATP production after LPS stimulation, and impaired glycolysis was shown to profoundly reduce the clearance of bacteria and fungi [56,57]. In contrast to M1 macrophages, activated M2 macrophages were reported to display STAT6− and PGC-1β-dependent decreases in glycolytic activity, while mitochondrial oxygen consumption rates and fatty acid oxidation were increased [58,59]. In line with the suggested M1 phenotype, stimulation of BMDMs with rFlaA:Betv1 resulted in a pronounced activation of HIF-1a signaling, which was not observed in BMDMs stimulated with equimolar amounts of rFlaA + rBet v 1. These results suggest the induction of HIF-1a signaling being critical for rFlaA:Betv1-mediated BMDM activation. Remarkably, succinate accumulating from a disrupted Krebs cycle in LPS-stimulated macrophages was shown to promote both glycolysis and IL-1ß production via the stabilization of HIF-1a (reviewed in [47]), thereby linking the activation of glycolytic metabolism to the activation of HIF-1a signaling. Interestingly, this newly discovered activation of HIF-1a signaling by rFlaA:Betv1 might further explain the previously observed induction of Th1-biased immune responses by flagellin:allergen fusion proteins in vitro and in vivo [15,16,17,18], as the activation of HIF-1a in macrophages was recently described to promote induction of Th1 responses [60]. Besides, M1 macrophages are known to produce pro-inflammatory cytokines, such as IL-12, TNF-α, IFN-γ, and IL-6, and can further induce Th1 responses [61]. The production of these cytokines was also reported to be downregulated in macrophage-specific HIF-1a knockout mice upon β-aminopropionitrile stimulation [62]. Indeed, we observed that rFlaA:Betv1 boosted production of the Th1 cytokine IFN-γ secretion even from BMDM:CD4^+^ T cell co-cultures with Th2-biased T cells (Figure 7).

### 4.4. MAPK, NF*κ*B, and mTOR Signaling Contribute to rFlaA:Betv1-Mediated BMDM Activation

Finally, we observed a pronounced activation of MAPK, NF*κ*B, and mTOR signaling in rFlaA:Betv1-stimulated BMDMs. Interestingly, the activation of these signaling pathways was TLR5-independent, while MAPK and NF*κ*B (but not mTOR) signaling were still observed in MyD88-deficient BMDMs.

We speculate MAPK signaling and mTOR/HIF-1a-controlled activation of BMDM metabolism to extensively cross-talk in rFlaA:Betv1-stimulated BMDMs. Here, activated p42/44 MAPK signaling can cross-activate mTOR signaling by either (i) activating PI3K [63,64,65,66,67], (ii) phosphorylation of TSC-2, which releases TSC-1/2-mediated mTOR inhibition [67,68], (iii) phosphorylation of regulatory associated protein of mTOR (RAPTOR), which promotes mTORC1 activation, and (iv) direct activation of the mTOR downstream target protein p70 S6 kinase [69,70,71].

In summary, the presented study reports the immune metabolic effects of a novel type of vaccine candidate generated by combining the TLR5 ligand flagellin with the major birch pollen allergen Bet v 1 (which serves a model antigen). Our results suggest that stimulation of BMDMs with rFlaA:Betv1 triggered a MyD88-dependent, but only partly TLR5-dependent, cytokine production, stronger activation of HIF-1a, MAPK, NF*κ*B, and mTOR signaling (with the activation of MAPK being located upstream of mTOR signaling), and increased glucose metabolism. In BMDM and CD4^+^ T cell co-cultures, rFlaA:Betv1 stimulation significantly suppressed Bet v 1-induced IL-5 and IL-13 secretion while inducing IFN-γ production (summarized in Figure 9). Therefore, macrophages contribute to the strong immune-modulating effects of rFlaA:Betv1 previously observed in vivo [15] and should be considered important target cells contributing to the re-establishment of allergen tolerance in AIT.

Finally, our results suggest that both the fusion of antigens to immune-activating molecules and the resulting structural alterations in the fused antigen can increase its immunogenicity, resulting in the induction of distinct immune responses. Therefore, such adjuvant:antigen fusion proteins likely are valuable tools to induce distinct (in our case balanced Th1) immune responses against antigens with otherwise either low immunogenicity or a high risk of triggering potentially harmful Th2 responses.

## Figures and Tables

**Table 1:** Primers used for cDNA synthesis.

Oligo Name	Sequence
NNSR_RT	gctcttccgatctctNNNNNN
NNSR_2	gctcttccgatctgaNNNNNN
NNSRnest_ind_N	CAAGCAGAAGACGGCATACGAGATNNNNNNGTGACTGGAGTTCAGACGTGTGCTCTTCCGATCTGA (N stands for a 6mer TruSeq index (Illumina))
NNSR_Illumina	AATGATACGGCGACCACCGAGATCTACACTCTTTCCCTACACGACGCTCTTCCGATCTCT

## Data Availability

The data presented in this study are available on request from the corresponding author.

## Supplementary Materials

The following are available online at https://www.mdpi.com/article/10.3390/cells10102614/s1: Figure S1: Both rFlaA and rFlaA:Betv1 activate BMDM metabolism, Figure S2: Activation of BMDM metabolism is accompanied by both pro- and anti-inflammatory cytokine secretion, Figure S3: The amount of LPS contained within the used rFlaA:Betv1 preparations do not induce BMDM activation, Figure S4: The used rFlaA:Betv1 preparation does not have mTLR2- or mTLR4-activating potential, Figure S5: Both rFlaA + rBet v 1 and rFlaA:Betv1 comparably reduce mitochondrial respiration, Figure S6: Toxicity of the tested inhibitors on C57BL/6 BMDMs, Figure S7: 96 h post-stimulation, rapamycin has only minor effects on rFlaA:Betv1-induced activation of BMDM metabolism and cytokine secretion, Figure S8: rFlaA:Betv1 induces a transcriptional shift towards both increased glycolytic metabolism and higher activation status in BMDMs.

## Abbreviations

2-DG	2-deoxy-glucose
ACO2	Aconitase 2
BMDM	Bone marrow-derived macrophage
CD	Cluster of differentiation
C/EBPβ	CCAAT enhancer-binding protein
ECAR	Extracellular acidification rate
GM-CSF	Granulocyte-macrophage colony-stimulating factor
IFN-γ	Interferon gamma
IL	Interleukin
Ipaf	ICE-protease activating factor
MAP(K)	Mitogen-activated protein (kinase)
(m)DC	(Myeloid) Dendritic cell
mTOR	Mammalian target of rapamycin
mTORC1	mTOR complex 1
MyD88	Myeloid differentiation primary response 88
NFκB	Nuclear factor ‘kappa-light-chain-enhancer’ of activated B-cells
OCR	Oxygen consumption rate
PI3K	Phosphatidylinositol 3 kinase
PDK:1	Pyruvate dehydrogenase kinase 1
PFK	Phospho-fructo-kinase
PFKFB3	6-phosphofructo-2-kinase/fructose-2,6-biphosphatase 3
PGM	Phosphoglycerate mutase
PKM	Pyruvate kinase isozyme
rBet v 1	Recombinant major birch pollen allergen number one from *Betula verrucosa*
rFlaA	Recombinant *Listeria monocytogenes* flagellin A
rFlaA:Betv1	Recombinant fusion protein of FlaA and Bet v 1
rmM-CSF	Recombinant mouse macrophage colony-stimulating factor
RAPTOR	Regulatory associated protein of mTOR
TSC	Tuberous sclerosis complex
Rot/AA	Rotenone/ antimycin A
SAP/JNK MAPK	Stress-activated protein kinase/c-Jun NH_2_-terminal kinase
Th1/2	T helper 1/2 cell
TLR	Toll-like receptor
TNF-α	Tumor necrosis factor alpha
