# Supplementary material for: The Flagellin:Allergen Fusion Protein rFlaA:Betv1 Induces a MyD88− and MAPK-Dependent Activation of Glucose Metabolism in Macrophages
Source: Cells. 2021 Oct 1;10(10):2614. doi: 10.3390/cells10102614 (PMC8534024; doi:10.3390/cells10102614)
Supplement: Supplementary file 1 [file cells-10-02614-s001.zip › cells-1288968 supplementary file.pdf]

cells

MDPI

Article

The Flagellin:Allergen Fusion Protein rFlaA:Betv1 Induces a MyD88− and MAPK-Dependent Activation of Glucose Metabolism in Macrophages

Yen-Ju Lin [1,†], Garibald Papp [1,†], Csaba Miskey [2], Anna Fiedler [1], Alexandra Goretzki [1], Sonja Wolfheimer [1], Jennifer Zimmermann [1], Peter Crauwels [3], Zoltán Ivics [2], Ger van Zandbergen [3,4,5], Stefan Vieths [1], Stephan Scheurer [1] and Stefan Schülke [1,*]

[1] Vice Presidents Research Group 1: Molecular Allergology, Paul-Ehrlich-Institut, 63225 Langen, Germany; Yen-Ju.Lin@pei.de (Y.-J.L.); gpapp@students.uni-mainz.de (G.P.); A.Malczyk@gmx.de (A.F.); Alexandra.Goretzki@pei.de (A.G.); Sonja.Wolfheimer@pei.de (S.W.); Jennifer.Zimmermann@pei.de (J.Z.), Stefan.Vieths@pei.de (S.V.), Stephan.Scheurer@pei.de (S.S.)

[2] Medical Biotechnology, Paul-Ehrlich-Institut, 63225 Langen, Germany; Csaba.Miskey@pei.de (C.M.); Zoltan.Ivics@pei.de (Z.I.)

[3] Immunology, Paul-Ehrlich-Institut, 63225 Langen, Germany; petcrauwels@hotmail.com (P.C.); Ger.vanZandbergen@pei.de (G.v.Z.)

[4] Institute of Immunology, University Medical Center of the Johannes Gutenberg University of Mainz, 55122 Mainz, Germany

[5] Research Center for Immunotherapy (FZI), University Medical Center, Johannes Gutenberg-University Mainz, 55122 Mainz, Germany

* Correspondence: Stefan.Schuelke@pei.de; Tel.: +49-6103-77-5209

† These authors equally contributed to this work.

Citation: Lin, Y.-J.; Papp, G.; Miskey, C.; Fiedler, A.; Goretzki, A.; Wolfheimer, S.; Zimmermann, J.; Crauwels, P.; Ivics, Z.; van Zandbergen, G.; et al. The Flagellin: Allergen Fusion Protein rFlaA: Betv1 Induces a MyD88− and MAPK-Dependent Activation of Glucose Metabolism in Macrophages. *Cells* **2021**, *10*, 2614. https://doi.org/10.3390/cells10102614

Academic Editors: Constantinos Pitsios and Caterina Chliva

Received: 22 June 2021
Accepted: 21 September 2021
Published: 1 October 2021

Publisher's Note: MDPI stays neutral with regard to jurisdictional claims in published maps and institutional affiliations.

Copyright: © 2021 by the authors. Licensee MDPI, Basel, Switzerland. This article is an open access article distributed under the terms and conditions of the Creative Commons Attribution (CC BY) license (http://creativecommons.org/licenses/by/4.0/).

Supplementary information

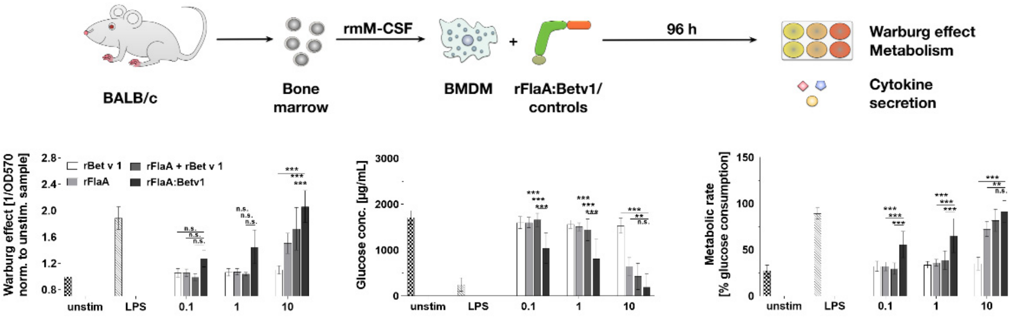

Repository Figure S1. Both rFlaA and rFlaA:Betv1 activate BMDM metabolism. BMDMs were differentiated from BALB/c bone marrow and stimulated with the indicated equimolar protein amounts or LPS as a positive control for 96 h. Supernatants were analyzed for the induced Warburg effect, glucose consumption from the culture medium as well as changes in metabolic rate. Data are mean results of three independent experiments ±SD with two technical replicates per experiment.

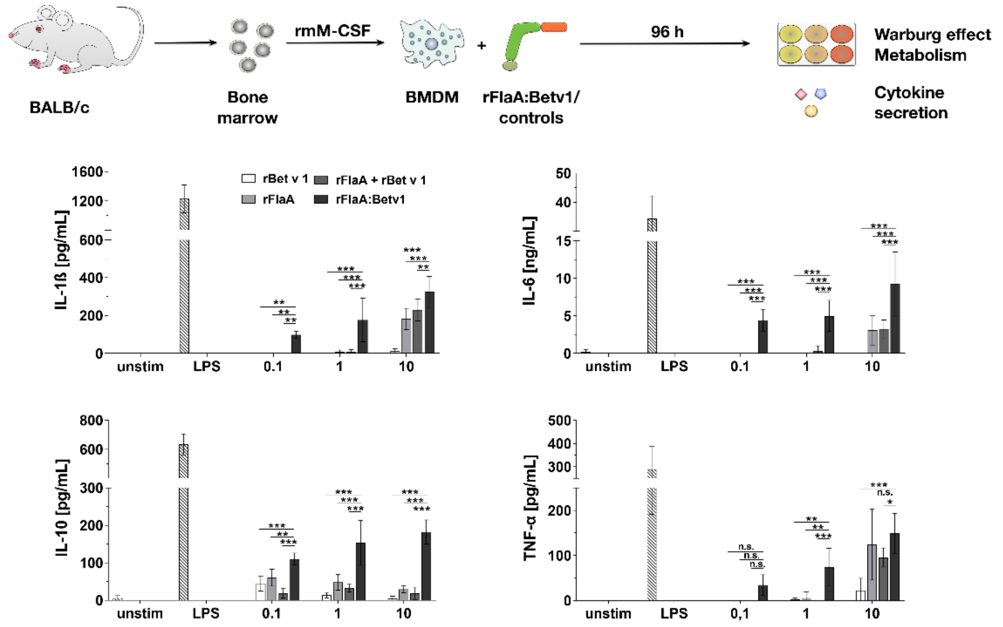

Repository Figure S2. Activation of BMDM metabolism is accompanied by both pro- and anti-inflammatory cytokine secretion. BMDMs were differentiated from BALB/c bone marrow and stimulated with the indicated equimolar protein amounts or LPS as a positive control for 96 h. Supernatants were analyzed for cytokine secretion by ELISA. Data are mean results of three independent experiments ±SD with two technical replicates per experiment.

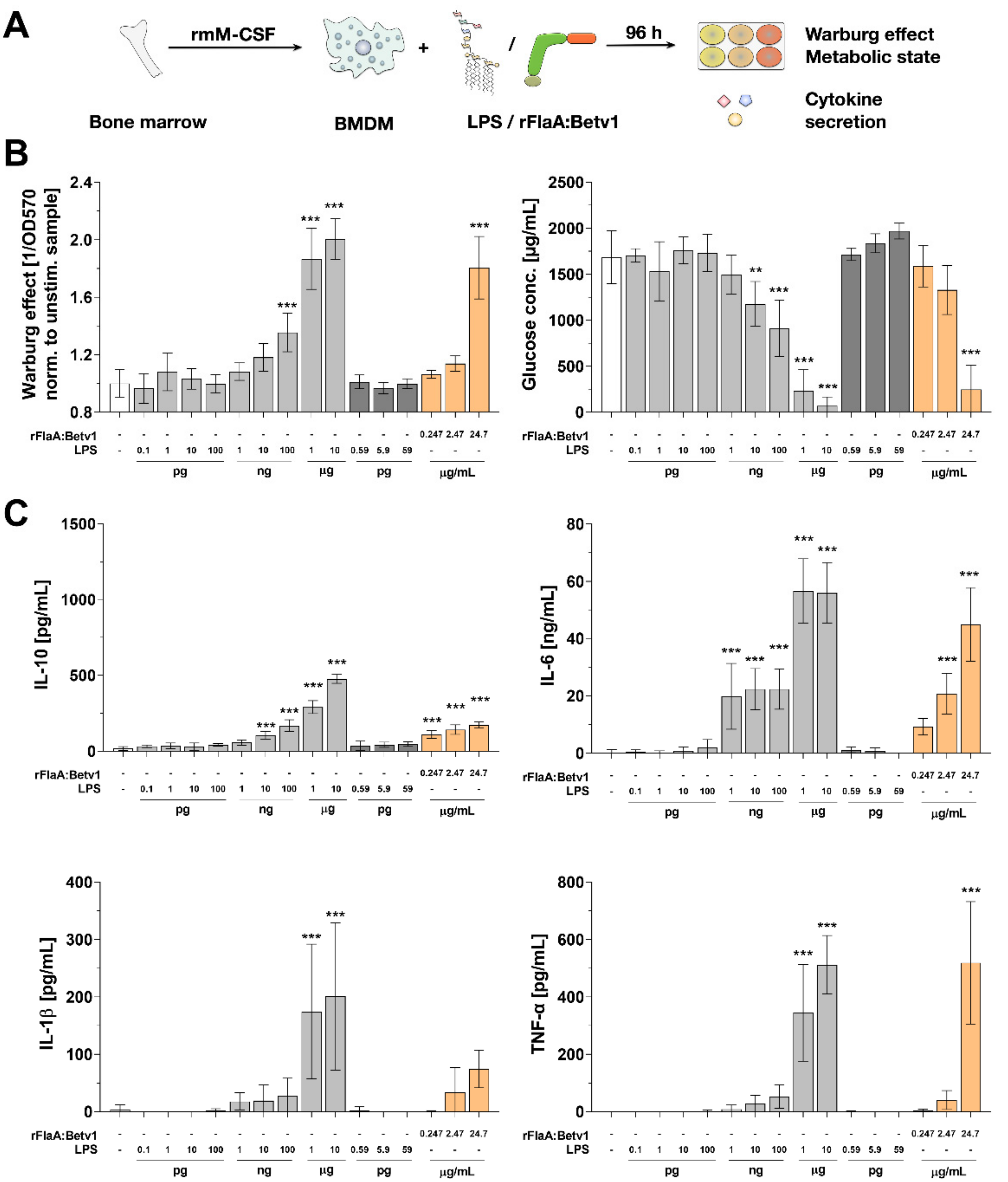

Repository Figure S3. The amounts of LPS contained within the used rFlaA:Betv1 preparations do not induce BMDM activation**.** C57BL/6 BMDMs were differentiated from mouse bone marrow and stimulated with the indicated amounts of either LPS to establish dose-response curves (light grey) the residual amounts of LPS contained within the applied concentrations of rFlaA:Betv1 (dark grey) to exclude BMDM activating effects, or rFlaA:Betv1 (orange) for 96 h (**A**). Supernatants were analyzed for the induced Warburg effect and glucose consumption (**B**) as well as cytokine secretion by ELISA (**C**). Data are mean results of three independent experiments±SD with two technical replicates per experiment.

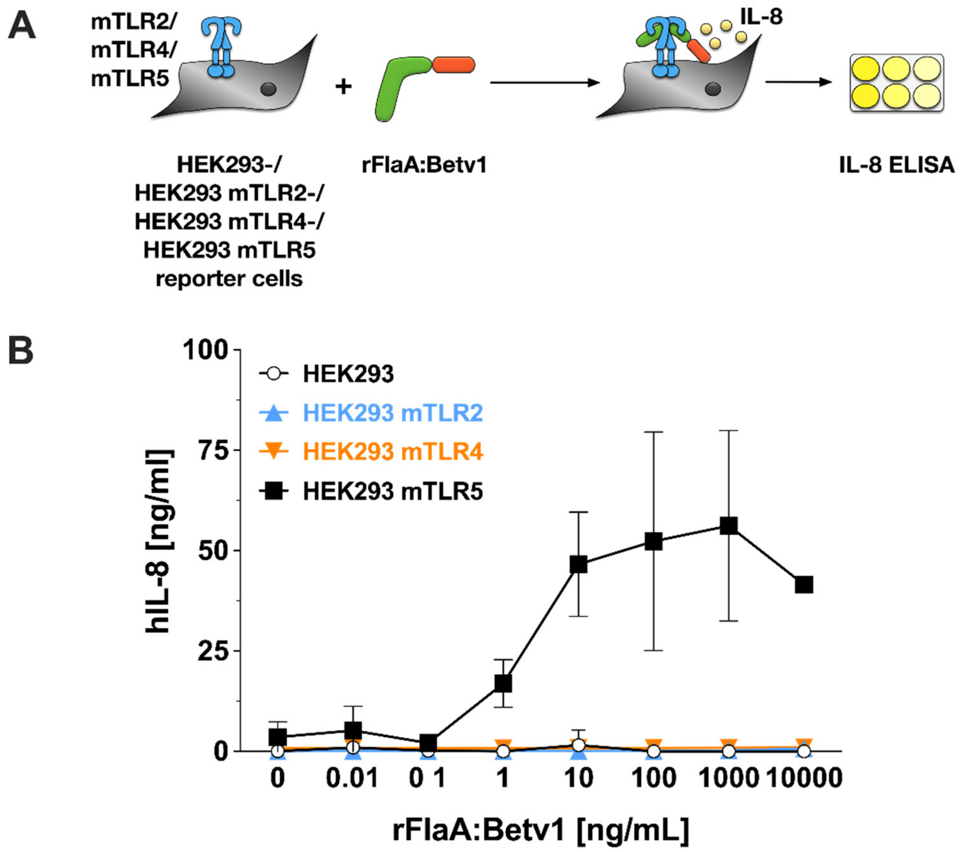

Repository Figure S4: The used rFlaA:Betv1 preparation does not have mTLR2- or mTLR4-activating potential. HEK293 cells stably transfected with either mouse TLR2, TLR4, or TLR5 were stimulated for 22 h with the indicated amounts of rFlaA:Betv1 (**A**). Non-transfected HEK293 cells were used as controls. IL-8 secretion into the culture supernatants was determined by ELISA (**B**). Data are mean of three independent experiments±SD with two technical replicates per experiment.

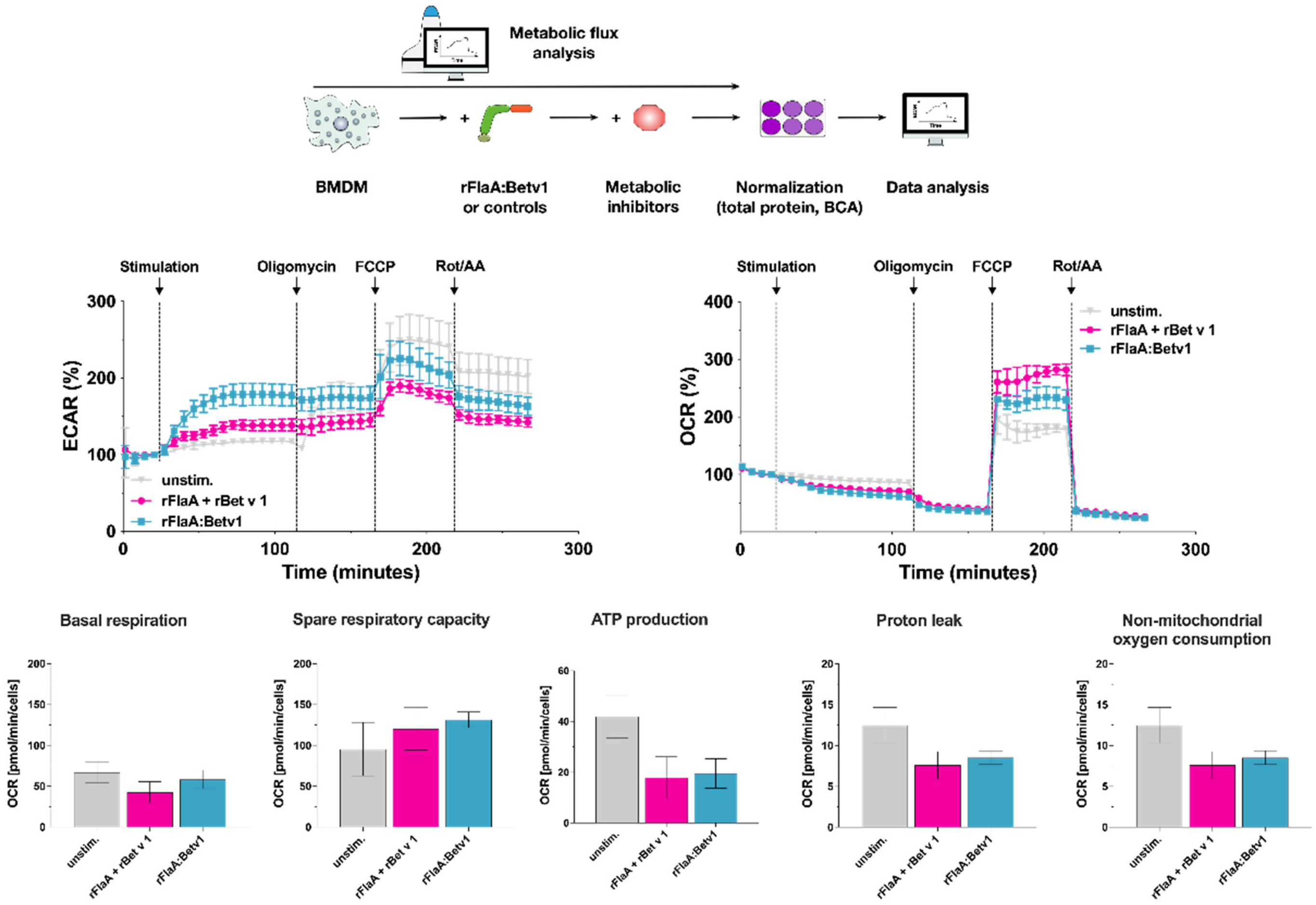

Repository Figure S5. Both rFlaA + rBet v 1 and rFlaA:Betv1 comparably reduce mitochondrial respiration. Mitochondrial function in either rFlaA + rBet v 1- or rFlaA:Betv1-stimulated BMDMs (both equimolar to 4 µg of rBet v 1) was analyzed by Seahorse Technology. Basal respiration, spare respiratory capacity, mitochondrial ATP production, proton leak, and non-mitochondrial respiration were analyzed using the “XF cell Mito Stress Test Report Generator” according to the manufacturer’s recommendations. Data are representative results of three independent experiments (with three to four technical replicates per experiment) that showed similar results.

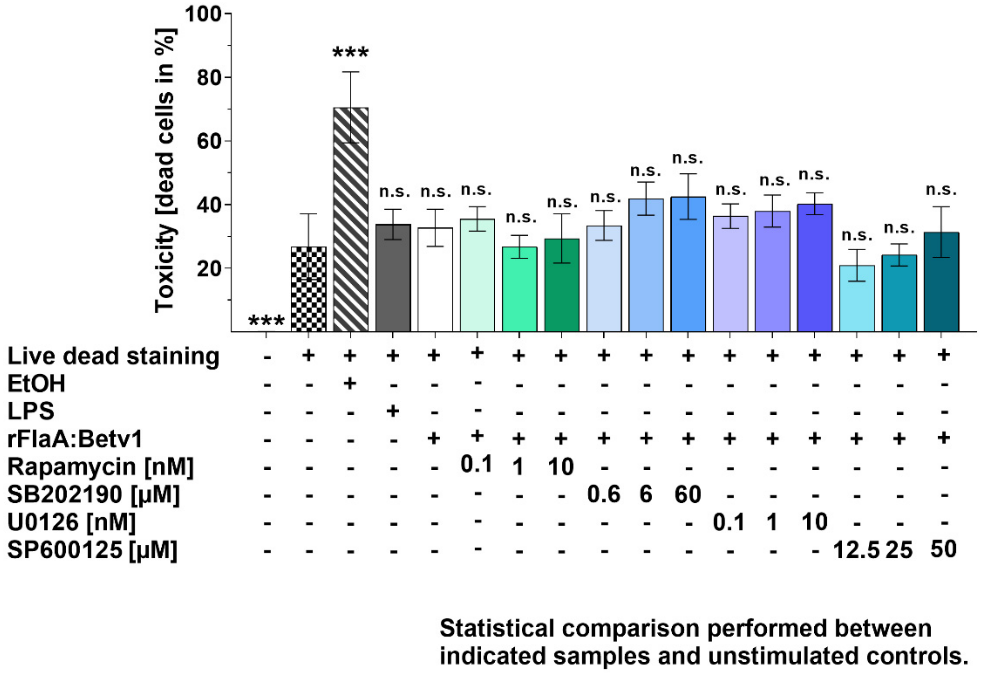

Repository Figure S6. Toxicity of the tested inhibitors on C57BL/6 BMDMs. C57BL/6 BMDMs were differentiated from bone marrow and stimulated with the indicated inhibitor concentrations, LPS, or rFlaA:Betv1, for 24 h. Cells killed by incubation with 70% EtOH served as positive controls. The frequency of dead cells was determined by live dead staining using eBioscience Fixable Viability Dye eFlour780 as recommended by the manufacturer. Data are mean results of three independent experiments ±SD with two technical replicates per experiment.

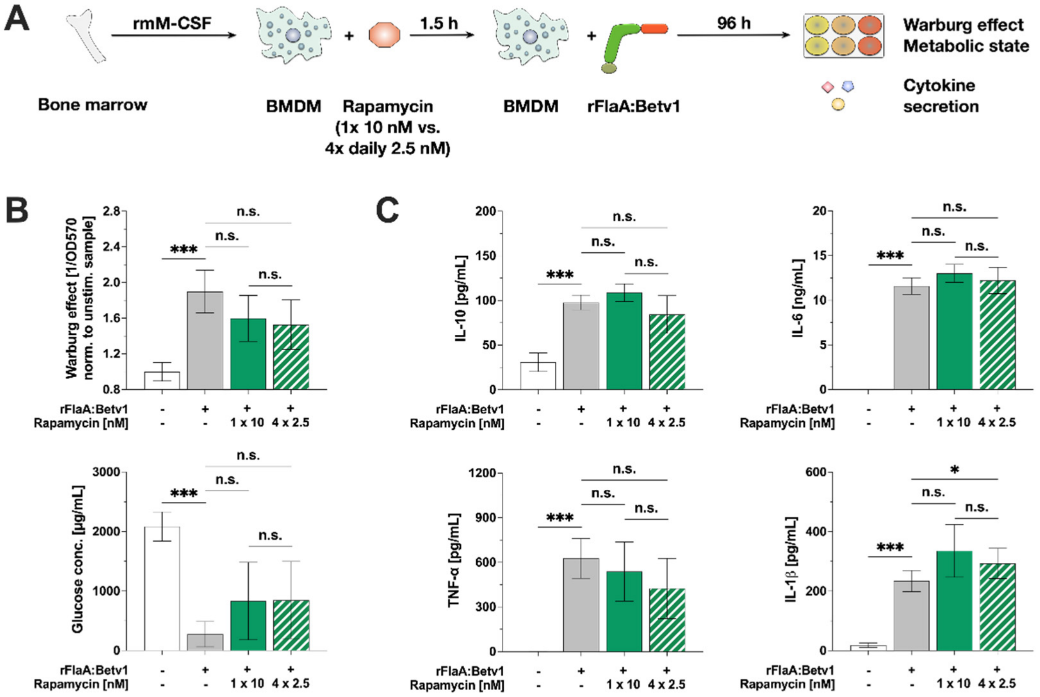

Repository Figure S7. 96 h post stimulation rapamycin has only minor effects on rFlaA:Betv1-induced activation of BMDM metabolism and cytokine secretion. C57BL/6 BMDMs were differentiated from bone marrow, pre-treated with either 10 nM rapamycin (mTOR inhibitor) once for 90 min or 2.5 nM rapamycin every 24 h, and subsequently stimulated with rFlaA:Betv1 for additional 96 h

(**A**). Cells were analyzed for their metabolic state (**B**) and cytokine secretion by ELISA (**C**). Data are mean results of three independent experiments±SD with two technical replicates per experiment.

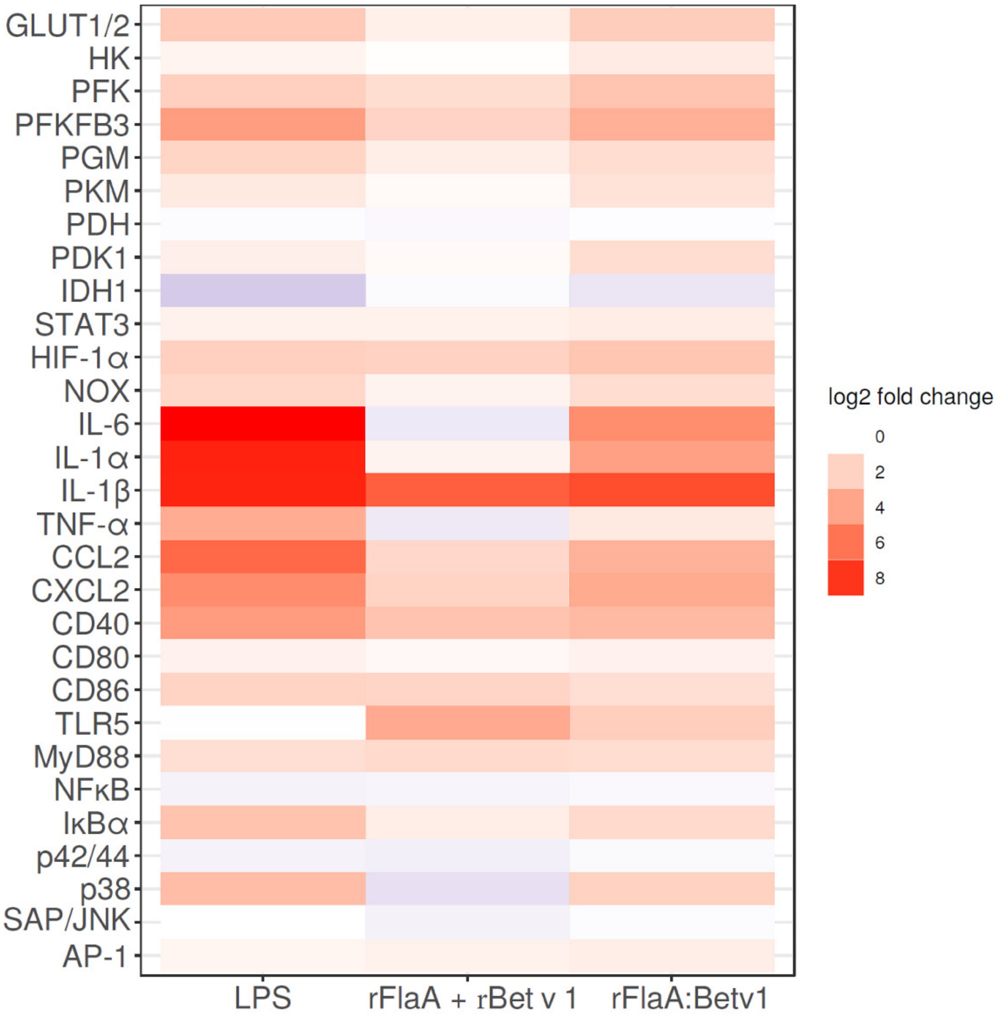

Repository Figure S8 rFlaA:Betv1 induces a transcriptional shift towards both increased glycolytic metabolism and a higher activation status in BMDMs. C57BL/6 BMDMs were differentiated from bone marrow and stimulated with LPS as positive control or equimolar amounts of either rFlaA + rBet v 1 or rFlaA:Betv1 for 48 h. Cells were harvested, used for RNA Seq-analyses, and mRNA-expression levels of the indicated molecules compared to the mean of unstimulated BMDMs were analyzed from the different stimulation conditions. Fold changes (log_2 scale) in mRNA expression level compared to unstimulated controls are indicated by color. Shown are mean expressional changes, obtained with four biological replicates per condition.
